# Ionic Liquids—A Review of Their Toxicity to Living Organisms

**DOI:** 10.3390/ijms22115612

**Published:** 2021-05-25

**Authors:** Ana R.P. Gonçalves, Xavier Paredes, A. F. Cristino, F. J.V. Santos, Carla S.G.P. Queirós

**Affiliations:** Centro de Química Estrutural, Faculdade de Ciências, Universidade de Lisboa, 1749-016 Lisbon, Portugal; raquelpgoncalves95@hotmail.com (A.R.P.G.); xpmendez@ciencias.ulisboa.pt (X.P.); afcristino@ciencias.uliboa.pt (A.F.C.); fjsantos@ciencias.ulisboa.pt (F.J.V.S.)

**Keywords:** Ionic liquids, toxicity, antimicrobial activity, ecotoxicity

## Abstract

Ionic liquids (ILs) were initially hailed as a green alternative to traditional solvents because of their almost non-existent vapor pressure as ecological replacement of most common volatile solvents in industrial processes for their damaging effects on the environment. It is common knowledge that they are not as green as desired, and more thought must be put into the biological consequences of their industrial use. Still, compared to the amount of research studying their physicochemical properties and potential applications in different areas, there is a scarcity of scientific papers regarding how these substances interact with different organisms. The intent of this review was to compile the information published in this area since 2015 to allow the reader to better understand how, for example, bacteria, plants, fish, etc., react to the presence of this family of liquids. In general, lipophilicity is one of the main drivers of toxicity and thus the type of cation. The anion tends to play a minor (but not negligible) role, but more research is needed since, owing to the very nature of ILs, except for the most common ones (imidazolium and ammonium-based), many of them are subject to only one or two articles.

## 1. Introduction

Ionic liquids (more accurately room-temperature ionic liquids (RTILs)) can be defined as materials composed of organic or inorganic cations (like imidazolium or pyridinium) and anions (e.g., nitrate, acetate, tetrafluoroborate, dicyanamide, bis(trifluoromethylsulfonyl)imide and lactate) that are liquid at or below 100 °C. The number of possible combinations of a cation and anion is estimated to reach 10^6^, making it theoretically possible to synthesize an IL targeted for a specific application or property [1]. ILs have been widely studied due to their unique properties, such as low flammability, negligible vapor pressure, high ionic conductivity, extensive liquid temperature range, high thermal and chemical stability, high solvation ability for organic, inorganic and organometallic compounds, selectivity and being easy recyclable through the separation from volatile compounds [2,3,4,5,6,7,8]. Due to these properties, ILs can be used in various areas from chemistry to engineering through the medical and the pharmaceutical fields, mainly as promising “green” alternatives to traditional organic solvents [9,10,11,12,13,14,15,16,17,18].

Initially, ILs were considered green solvents, safe for the environment and human life and health, mainly due to their negligible vapor pressure. However, some ILs are produced from non-renewable energy sources and are poorly biodegradable in the environment, undermined their green character [19,20,21]. When designing and synthesizing new chemical products as suggested in the 3rd, 4th and 10th green chemistry principles, not only their efficiency and cost should be considered, but also their toxicity to human health and environment [22,23]. ILs are not currently widely used for commercial purposes. However, some companies have started their industrial use, BASIL™ from BASF probably being the most widely known. Consequently, for a larger production (over one metric ton per year), safety information on the ILs must be provided, according to current legislation, like, for example, the European Union Regulation REACH demand [24].

**Figure 1:**
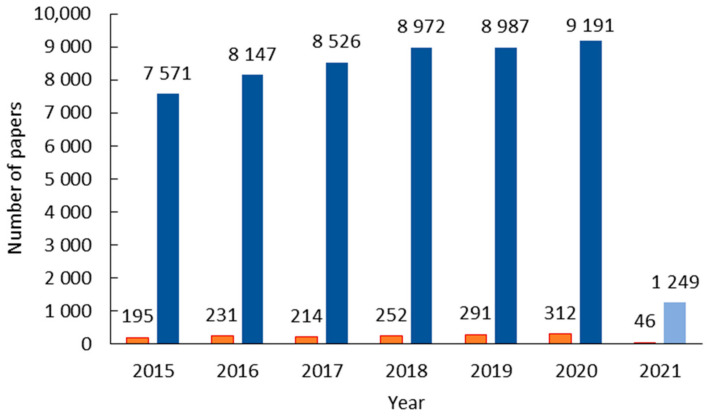
Number of scientific papers published from 2015 to 15th March 2021 concerning “ionic liquids” (blue columns) and “toxicity ionic liquids” (orange columns).

The disposal of ILs is mainly performed on wastewater discharges and leaching of landfills leading to increased concern about contaminating aquatic and terrestrial ecosystems. The first studies concerning the toxicology of ILs began in the 2000s by Pernak and collaborators [25,26], where the authors analyzed the antimicrobial activity of pyridinium, bispyridinium and benzimidazolium chlorides. In 2015, Amde et al. [27] published a review where the transport and transformation of ILs in the environmental systems were studied and a compilation and analysis of the existing data about the toxicity of ILs. The authors presented a scheme resuming how structural modifications (cation type, cation side-chain and anion effect) influence the toxicity of ILs. They refer that cholinium-based ILs are the least toxic ILs, that the increase of the side-chain hydrophobicity and its length increases the toxicity and the increase of anion lipophilicity and instability also increases the ILs toxicity. The authors also present some routes for synthesizing or selecting the less toxic and/or more biodegradable IL. A year later, a meta-analysis of ionic liquid literature and toxicology [28] was published where the authors referred that from the total amount of publications of ILs, only less than 3% were studies concerning their toxicity. A percentage that tracks well with Pawlowska et al. [21] showing that in 2018 the number of publications (in the Web of Science database) dealing with ILs was more than 8000, and for the keywords “toxicity ionic liquid,” only 250 were retrieved. This number is significantly low compared to the vast number of ILs that can be tailored (Figure 1). Another important issue is related to the effect of ILs on different organisms. For example, an IL can be toxic to an organism but practically harmless to another, so it is important to analyze the toxic effect of ILs on different organisms, from the simpler ones (bacteria or fungi) to the more complex (plants and animals).

The need generated by the project ILGerants—new ionic liquids-based mixtures for absorption refrigeration (http://ilgerants.rd.ciencias.ulisboa.pt/, access date: 16 March 2021) in choosing the most appropriate ILs for absorption refrigeration, while considering their toxicity, led to this review, whose purpose is to condense all of the most recent toxicological (plants, microorganisms and animal) studies of ILs from 2015 onwards. Still, it is important to refer that besides toxicity, the biodegradability of ILs is also an important environmental factor and should also be studied.

## 2. Effect of Ionic Liquids on Microorganisms

In life sciences and medicine, one of the most sought-after characteristics of ionic liquids is their antimicrobial and antifungal activities [25,26,29]. Therefore, the antimicrobial and antifungal activities of ILs could be beneficial and a great development in some medical applications. However, with such properties also comes along the impact in unicellular organisms that play an important role in the balance of the ecosystems. The persistence of ILs in the environment may disturb the role those microorganisms play in the balance of ecosystems, motivating new designs using amino acids and choline-based ILs to try to overcome the threat they pose to the environment by lowering their toxicity [30,31]. The microorganisms most commonly used in the ILs toxicity research are the bacteria *Aliivibrio fischeri* (*A. fischeri*), *Escherichia coli* (*E. coli*), *Pseudomonas putida* (*P. putida*), *Staphylococcus aureus* (*S. aureus*) and *Aeromonas hydrophila* (*A. hydrophila*), and the yeast *Candida albicans* (*C. albicans*) and *Aspergillus niger* (*A*. *niger*).

### 2.1. Bacteria

The most studied bacteria are *Aliivibrio* *fischeri* (*A. fischeri)*, a Gram-negative bioluminescent marine bacterium. The most common toxicological bioassay used in the aquatic field is the *A. fischeri* bioluminescence assay. This test has an easy interpretation, high sensibility and a good relation speed/cost compared to other assays. This test relies on the relationship between the cellular metabolism of *A. fischeri* and its luminescence, thus allowing to relate decreased light intensity to the toxicity it is exposed to.

With the purpose of developing a new method based on the *A. fischeri* assay, Costa et al. [32] study the toxicological effect of seven ILs (1-ethyl-3-methylimidazolium methanesulfonate [C_2_mim][CH_3_SO_3_]; 1-ethyl-3-methylimidazolium bis(trifluoromethylsulfonyl)imide [C_2_mim][(CF_3_SO_2_)_2_N]; 1-ethyl-3-methylimidazolium acetate [C_2_mim][CH_3_COO]; choline acetate [Cho][CH_3_COO]; 1-butyl-4-methylpyridinium chloride [C_4_mpy][Cl]; 1-butyl-4-methylpyridinium tetrafluoroborate [C_4_mpy][BF_4_] and tetrabutylammonium chloride [N_4,4,4,4_][Cl]) in this bacterium. The authors found that the toxicity was higher for the ILs with aromatic rings incorporated in the cation core relatively to ILs with non-aromatic groups. When comparing the ILs with same anion ([C_2_mim][CH_3_COO] vs. [Cho][CH_3_COO] and [C_4_mpy][Cl] vs. [N_4,4,4,4_][Cl]), the ILs with non-aromatic cations presented higher EC_50_ values than the ones with aromatic cations. In addition, the imidazolium-based ILs presented a lower toxicity than the pyridinium-based ILs. Regarding the anion, it was observed that the acetate and methanesulfonate anions were the less toxic to *A. fischeri* and that the tetrafluoroborate anion was the more toxic. The order of toxicity observed in this study was: [C_4_mpy][BF_4_] (EC_50_ = 45.3 mg·L^−1^) > [C_4_mpy][Cl] (EC_50_ = 164 mg·L^−1^) > [N_4,4,4,4_][Cl] (EC_50_ = 400 mg·L^−1^) > [C_2_mim][(CF_3_SO_2_)_2_N] (EC_50_ = 837 mg·L^−1^) > [C_2_mim][CH_3_COO] (EC_50_ = 1637 mg·L^−1^) > [Cho][CH_3_COO] (EC_50_=1843 mg·L^−1^) > [C_2_mim][CH_3_SO_3_] (EC_50_ = 14,083 mg·L^−1^).

Montalbán and coworkers [33] measure the toxicity of twenty-nine (imidazolium, pyridinium and ammonium-based) ILs on *A. fischeri* by Microtox^®^ (the most used commercial kit to evaluate the toxicity of compounds against *A. fischeri*). As previously mentioned, pyridinium-based ILs present a higher toxicity than the similar imidazolium-based ILs, as shown by two pairs of ILs with the same alkyl chain and anion: [C_4_mpy][BF_4_] (log EC_50_ = 2.44 μM) vs. 1-butyl-3-methylimidazolium tetrafluoroborate ([C_4_mim][BF_4_], log EC_50_ = 3.46 μM) and 1-ethyl-3-methylpyridinium ethylsulfate ([C_2_mpy][C_2_H_5_OSO_3_], log EC_50_ = 3.83 μM) vs. 1-ethyl-3-methylimidazolium ethylsulfate ([C_2_mim][C_2_H_5_OSO_3_], log EC_50_ = 4.10 μM). In this work, the effects of alkyl chain length and the anion on toxicity were also studied. The toxicity was higher for the ILs with longer alkyl chain length: 1-octyl-3-methylimidazolium hexafluorophosphate ([C_8_mim][PF_6_], log EC_50_ = 1.25 μM) > 1-hexyl-3-methylimidazolium hexafluorophosphate ([C_6_mim][PF_6_], log EC_50_ = 2.24 μM) > 1-butyl-3-methylimidazolium hexafluorophosphate ([C_4_mim][PF_6_], log EC_50_ = 3.32 μM) > 1-ethyl-3-methylimidazolium hexafluorophosphate ([C_2_mim][PF_6_], log EC_50_ = 4.22 μM). The ILs with anions containing fluorine atoms presented higher toxicity, and their toxicity increased with the increase in the number of fluorine atoms. The order in IL toxicity for the different anions found was: [(CF_3_SO_2_)_2_N] > [PF_6_] > [BF_4_] ≈ methylsulfate ([CH_3_OSO_3_]) ≈ 2-(2-methoxyethoxy)ethylsulfate ([MDEGSO_4_]) > trifluoromethanesulfonate ([CF_3_SO_3_]) > [CH_3_COO] > [C_2_H_5_OSO_3_] > [Cl]. Similar work was performed by Delgado-Mellado et al. [34] and Kusumahastuti et al. [35]. The first workshop focused on the ecotoxicity of 24 imidazolium- and pyridinium-based ionic liquids towards *A. fischeri* also concluding that the pyridinium-based ILs lead to higher toxicity and that the cation alkyl chain length had the major influence on the IL toxicity, while the anion played a minor role. The second work studied the toxicity of 24 L-phenylalanine-derived ILs. To evaluate the effect of the alkyl chain length, the authors used an L-phenylalanine moiety as a base with two side-chains, one of them with pyridinium, imidazolium or cholinium; the other with an alkyl chain of 2 to 16 carbon atoms (C_2_–C_16_). The authors verified that the alkyl chain length was responsible for the ILs toxicity and that all ILs with alkyl chains bigger than C_10_ were toxic. However, the toxicity from C_10_ to C_14_ was almost constant, with a slight decrease when the alkyl group was extended to C_16_.

**Figure 2:**
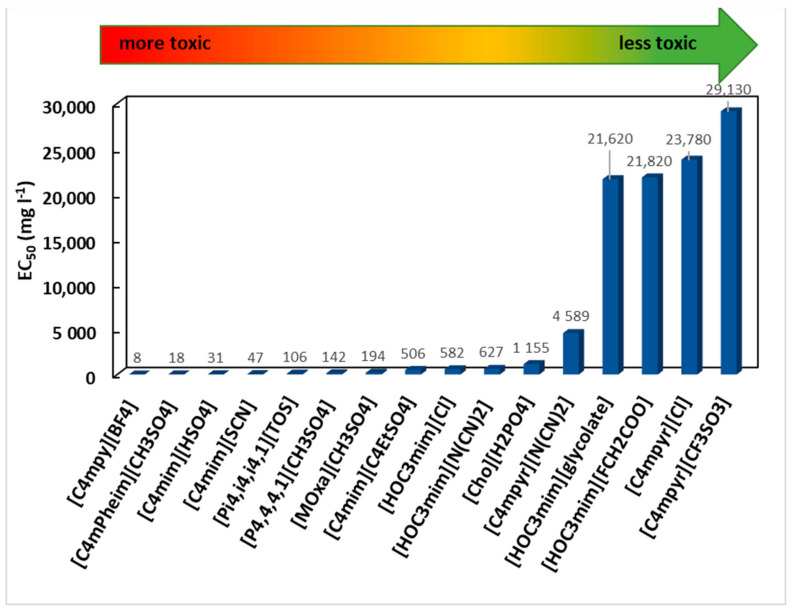
EC_50_ values for the ILs towards *A. fischeri* studied by Hernández-Fernández et al. [36].

Hernández-Fernández et al. [36] presented the toxicity data of sixteen ILs, also determined by the *A. fischeri* bioassay, to obtain qualitative structure–property relationships to establish a guideline for designing “greener” ILs. Based on cation composition, the pyrrolidinium-based ILs ([C_4_mpyr][Cl] EC_50_ > 23,780 mg·L^−1^, [C_4_mpyr][N(CN)_2_] EC_50_ = 4588.85 mg·L^−1^ and [C_4_mpyr][CF_3_SO_3_] EC_50_ > 29,130 mg·L^−1^) presented the lowest toxicity values. The imidazolium and pyridinium-based ILs were found to be the most toxic [C_4_mpy][BF_4_] (EC_50_ = 7.60 mg·L^−1^) and 1-butyl-3-methyl-2-phenylimidazolium methylsulfate ([C_4_mPheim][CH_3_OSO_3_], EC_50_ = 17.83 mg·L^−1^). The most hydrophobic character of the imidazolium and pyridinium cations seems to explain their higher toxicity since it increases the possibility of their interaction with cell membranes. The authors also observed that incorporating a hydroxyl group in the alkyl substituent of the imidazolium ring ([HOC_3_mim]) significantly decreases the toxicity of imidazolium-based ILs ([C_4_mim]). The toxicity order of the ILs studied is presented in Figure 2.

Other work using *A. fischeri* was the one published by Ghanem et al. [37], where after the synthesis and characterization of four amino acid-derived ILs (AAILs), their toxicologic effect towards a green algae and *A. fischeri* was studied. The AAILs synthesized were: 1-(2-hydroxyethyl)-3-methylimidazolium glycinate ([OHC_2_mim][Gly]), 1-(2-hydroxyethyl)-3-methylimidazolium alaninate ([OHC_2_mim][Ala]), 1-(2-hydroxyethyl)-3-methylimidazolium serinate ([OHC_2_mim][Ser]) and 1-(2-hydroxyethyl)-3-methylimidazolium prolinate ([OHC_2_mim][Pro]). The [OHC_2_mim][Ala] presented a higher toxicity value compared with the others AAILs. The value of EC_50_ decreased in the following order: [OHC_2_mim][Pro] (EC_50_ = 14,509.36 ± 0.005 mg·L^−1^) > [OHC_2_mim][Gly] (EC_50_ = 11,469.23 ± 0.003 mg·L^−1^) > [OHC_2_mim][Ser] (EC_50_ = 10,526.46 ± 0.004 mg·L^−1^) > [OHC_2_mim][Ala] (EC_50_ = 8123.27±0.005 mg·L^−1^), also all the AAILs can be classified as harmless.

The *A. fischeri* bioassay was also used in the toxicological study of several magnetic ionic liquids in the work of Sintra and coworkers [38]. This work had as objective to evaluate the effect of different anions on cholinium-based ILs ([N_1,1,1,2(OH)_] corresponds to the cholinium cation). However, the ILs that presented the lowest and highest toxicity were the ones with the [MnCl_4_] anion, the [N_1,6,2(OH),2(OH)_]_2_[MnCl_4_] (EC_50_=69.54 mg L^−1^) and the [N_1,1,12,2(OH)_]_2_[MnCl_4_] (EC_50_=0.76 mg L^−1^), indicating once more that the cation influences the toxicity more than the anion. The effect of the alkyl chain length was also observed for this type of ILs. The effect of the metal anion was studied, comparing them with the results obtained for the bromide anion ([Br]), where the replacement of this anion by a metal one originated a significant increase in the toxicity. Based on the EC_50_ values, [N_1,1,1,2(OH)_]_2_[CoCl_4_] (EC_50_ = 8.90 mg L^−1^), [N_1,1,12,2(OH)_][FeCl_4_] (EC_50_ = 1.04 mg L^−1^), [N_1,1,12,2(OH)_]_2_[MnCl_4_] (EC_50_ = 0.76 mg L^−1^), [N_1,1,12,2(OH)_]_2_[CoCl_4_] (EC_50_ = 7.84 mg L^−1^) and [N_6,2(OH),2(OH),2(OH)_][FeCl_4_] (EC_50_ = 5.99 mg L^−1^) are classified as toxic and all the others are classified as moderately toxic (see Table 1).

The toxicity of the ILs towards *A. fischeri* was also studied using quantitative structure–activity relationship (QSAR) models. Das et al. [39] developed predictive QSAR models for the toxicity of ILs on *A. fischeri* to determine the toxicity of new ILs and fill in the existing toxicity data gap. Yan and coworkers [40] also presented an accurate QSAR model for predicting the *A. fischeri* toxicity based on a topological method. The multiple linear regression method developed had a correlation coefficient (*R*^2^) = 0.908 with an average absolute error of 0.278, using a data set of 157 ILs. Wang et al. [41] determined the toxicities of twenty-four bromide-based ILs towards *A. fischeri* and *Daphnia magna* (a small planktonic crustacean) and developed QSAR models for these two organisms. The results showed that the size of the cation mainly determined the toxicity and that it would increase with the length of the alkyl side-chains independently of the type of cation and, for the same alkyl side-chain length (excluding the octyl side-chain), the toxicity order was: pyridinium > imidazolium > piperidinium > pyrrolidinium. In the case of *Daphnia magna,* the toxicity order was imidazolium > pyridinium > piperidinium > pyrrolidinium. The methods developed had an *R*^2^ = 0.954 and *R*^2^ = 0.895 for *A. fischeri* and *Daphnia magna*.

Besides the tests with *A. fischeri*, other bacteria were also studied mainly to understand the difference in behaviors of Gram-positive and Gram-negative bacteria in the presence of ILs. Mester and coworkers [42] performed a toxicological profile of ILs towards Gram-positive bacteria, *Listeria monocytogenes* (*L. monocytogenes*) and Gram-negative bacteria *Escherichia coli* (*E. coli*). The Gram-positive bacteria were more tolerant to ILs toxicity than the Gram-negative bacteria. However, the influence of the cation side-chain length and the anion were similar. The toxicity for the ILs with the same anion followed: [C_6_mim] > [C_4_mim] > [C_4_pyr] > [C_2_mim], proving once again that the increase of lipophilicity of the cation brings about a perturbation of biomembranes. It was also observed that the increase of the toxicity of ILs increases significantly more between [C_4_mim] and [C_6_mim] than between [C_2_mim] and [C_4_mim]. Considering the ILs with the same cation ([C_4_mim]), toxicity followed the tendency: tricyanomethanide [C(CN)_3_] > thiocyanate ([SCN]) ≈ [N(CN)_2_]" [Cl] > [CH_3_OSO_3_] and the [C_4_mim][CH_3_OSO_3_] demonstrated to be not toxic for *L. monocytogenes* for the duration of the experiment. The same authors also studied the capacity of strains of *L. monocytogenes* to adapt to high concentrations of thirteen ILs [43]. The results showed a significantly high tolerance of all *L. monocytogenes* strains possessing transposon Tn*6188*, mainly for imidazolium-based ILs with longer alkyl chain length. The main reason why the impact of Tn*6188* could only be observed for long alkyl chain length ILs, was because the toxicity of ILs correlates strongly with the length of the alkyl side-chain of the cation and probably not only the presence of Tn*6188* enables *L. monocytogenes* to withstand higher concentration of ILs, but also possibly a combination of different cellular stress responses, for example, cell wall modifications or accumulation of osmolytes. Another interesting behavior was that strains not only adapted to imidazolium-based ILs with longer alkyl chains length but also became more resistant compared to their parents’ strain. Although the authors suggest that more studies are needed, and the genetic background of bacteria should be considered in this type of study.

Yu et al. [44] investigated the antimicrobial activities of twelve piperazinium and guanidinium-based ILs against *E. coli* and *Staphylococcus aureus* (*S. aureus*). To evaluate the antimicrobial activity, the authors determined the minimum inhibitory concentration (MIC). MIC values of the ILs were higher against *S. aureus* than *E. coli*, except for 1-phenylpiperazinium tetrafluoroborate ([Phpi][BF_4_]) and 1-methylpiperazinium lactate ([C_1_pi][Lac]). The ILs [C_1_pi][Lac], 1-ethylpiperazinium lactate ([C_2_pi][Lac]) and 2-ethyl-1,1,3,3-tetramethylguanidinium ethylsulfate ([(C_2_)^2^(C_1_)_2_(C_1_)_2_^3^gu][C_2_H_5_OSO_3_]) presented MIC values higher than 100 mg·mL^−1^ for both bacteria and can be classified as nontoxic. Moreover, the ILs 2-ethyl-1,1,3,3-tetramethylguanidinium iodide ([(C_2_)^2^(C_1_)_2_(C_1_)_2_^3^gu][I]) and 2,2-diethyl-1,1,3,3-tetramethylguanidinium ethylsulfate ([(C_2_)_2_^2^(C_1_)_2_(C_1_)_2_^3^gu][C_2_H_5_OSO_3_]), against *S. aureus*, had also MIC values higher than 100 mg·mL^−1^ and can also be classified as nontoxic for this type of bacteria. It was also observed that the toxicity of ILs depends on the cation and anion (notable differences were detected between [I] and [C_2_H_5_OSO_3_]) and that the length of the alkyl chain plays an important role. The ILs with the [BF_4_] anion were relatively more toxic against both bacteria due to the release of fluorine from hydrolysis, which can inhibit the Na^+^-K^+^-ATP enzyme causing cell death. Through scanning electron microscopy (SEM), it was possible to observe the damage in the bacteria cell membrane and that *E. coli* was more susceptible to damage than *S. aureus*.

Borkowski et al. [45] observed the toxic effects of theophylline-based ILs (theophylline is also known as 3.7-dihydro-1,3-dimethyl-1H-purine-2,6-dione or 1,3-dimethylxanthine) towards *E. coli* (Gram-negative bacterium) and *Bacillus cereus* (*B. cereus*), a Gram-positive bacterium. The ILs studied were: dibenzyldimethylammonium theophyllinate, alkylbenzyldimethylammonium theophyllinate and oleyltrimethylammonium theophyllinate being the last 2 ILs mixtures of the same anion with at least two cations of the same type with different alkyl chain length (for more details, the reader is referred to the original article). The bacteria exhibited different sensitivity to the ILs studied in terms of growth, activity and viability, being the *B. cereus* the most sensitive. Even at the lowest concentrations, *B. cereus* cells could not reproduce in the presence of the last 2 ILs, presenting a substantial decline in respiration rate and dehydrogenase activity. Meanwhile, the *E. coli* cells were capable of growth and displayed significantly higher activity in the presence of the second IL, mainly at lower IL concentrations. However, these bacteria exhibited a considerable increase of activity in the last IL presence, but no microbial growth was observed. This means that besides this one that could inhibit the proliferation of the Gram-negative bacteria, they can maintain their metabolic activity. The authors also notice that the last 2 ILs caused notable damages to *E. coli* cell membranes. Still, the *B. cereus* cells did not exhibit remarkable changes, although in *B. cereus* was more notable the initial adverse effects related to inhibition and dysregulation of cellular metabolism. The effect of the cation alkyl chain length was also observed. To test if the toxicity of these ILs is dependent on the type of lipopolysaccharide (LPS) and type of bacteria, the same laboratory [46,47], studied the interaction and antimicrobial activity of ILs with the cation [N_1,1,1,n_] (n = 8, 10, 12, 14, 16, 18) and the theophyllinate anion on five different *E. coli* strains (K12, R1, R2, R3 and R4) and one Gram-positive strain, *B. cereus*. It was observed that these ILs and their precursors could interact with *E. coli* strains via a mechanism that appears to be dependent on the LPS composition. The cutoff effect of the alkyl chain was observed in the transition from C_16_ to C_18_. The length of the alkyl chain plays an important role in both interactions with the bacterial membrane and generation of oxidative stress, correlated with intracellular damage to DNA. The same authors deepened the study on the adaptation of *E. coli* to lethal concentrations of these theophylline-based ILs [48]. For the adaptation study, only the IL with C_14_ alkyl chain length was used, and apart from a quick adaptation to increased concentrations of this IL, it was also observed that the bacteria also became more resistant to the ILs with other alkyl chain lengths (C_12_-C_18_). It was also detected that these types of ILs modify the electrokinetic potential of bacterial membranes, giving rise to the aggregation of bacterial cells. The increase of alkyl chain length also caused increased membrane fluidity. Besides the alkyl chain length effect, the cutoff effect was observed, where the toxicity of octadecyltrimethylammonium theophyllinate (C_18_) decreased concerning hexadecyltrimethylammonium theophyllinate (C_16_) and tetradecyltrimethylammonium theophyllinate (C_14_). In this study, the less toxic ionic liquid was the octyldecyltrimethylammonium theophyllinate (C_8_), and the most toxic was the hexadecyltrimethylammonium theophyllinate (C_16_).

Nancharaiah et al. [49], studied the hormetic effect of three ILs ([C_2_mim][CH_3_COO], 1-ethyl-3-methylimidazolium diethylphosphate ([C_2_mim][DEP]) and 1,3-dimethylimidazolium dimethylphosphate ([C_1_mim][DMP])) against two bacteria, the Gram-positive *Clostridium* sp. and the Gram-negative *Pseudomonas putida* (*P. putida*). The authors observed that bacterial growth was enhanced (via pH regulation) for concentrations of [C_2_mim][CH_3_COO] up to 2.5 g·L^−1^ in a medium containing a fermentable organic substance where the IL acts as a buffer, and inhibition took place for higher concentrations. No significant effects were observed for concentrations of [C_2_mim][DEP] up to 2.5 g·L^−1^ and up to 4 g·L^−1^ of [C_1_mim][DMP], and again, retarded or inhibited growth occurring for higher concentrations.

**Figure 3:**
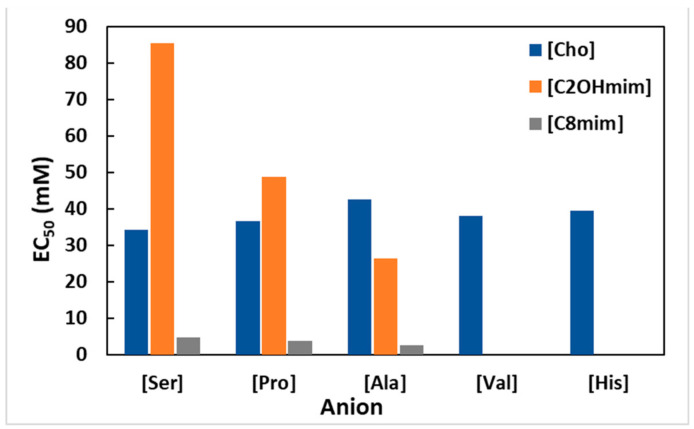
EC_50_ values for the ILs towards *A. fischeri* studied by Foulet et al. [50] and Ghanem et al. [37].

The antimicrobial activities of five choline-amino acids-based ILs (choline serinate ([Cho][Ser]), choline valinate ([Cho][Val]), choline prolinate ([Cho][Pro]), choline histidinate ([Cho][His]) and choline alaninate ([Cho][Ala])) against the Gram-positive bacterium, *Listeria monocytogenes* (*L. monocytogenes*) and the Gram-negative bacteria: *Aeromonas hydrophila* (*A. hydrophila*) and *Klebsiella pneumoniae* (*K. pneumoniae*) was performed by Foulet et al. [50]. All the choline-amino acids ILs showed to be relatively toxic against the microbial species studied. In addition to [Cho][Ser], all the other ILs had similar EC_50_ values. [Cho][Ser] presented the lowest antimicrobial activity against both Gram-negative bacteria, probably because L-serine has one more hydroxyl group than the others amino acids studied. It was also observed that *A. hydrophila* is less resistant than *K. pneumoniae* and *L. monocytogenes*. Comparing these results with the ones previously reported by Ghanem et al. [37], it can be seen that the cation is the main actor in the antimicrobial activity of these amino acid ILs. In the case of *L. monocytogenes* and the [Ser] and [Pro] anions, [OHC_2_mim] is less toxic than [Cho] and [C_8_mim] cations. For the [Ala] anion, the less toxic cation is the [Cho], followed by [OHC_2_mim] and [C_8_mim] is the most toxic (Figure 3). Meaning that, as important as the cation is in the toxicity of the IL, the anion is still relevant.

The toxicology of choline oleate ([Cho][Ol]) and choline laurate ([Cho][Lau]) was evaluated against four bacteria [51]: *E. coli*, *S. aureus*, *L. monocytogenes* and *A. hydrophila*. The results of these ILs against *E. coli* and *S. aureus* showed that they could be classified as practically harmless, although [Cho][Ol] (EC_50_ = 1.38 mM and EC_50_ = 1.59 mM, respectively) presented higher toxicity than [Cho][Lau] (EC_50_ = 16.47 mM and EC_50_ = 5.75 mM, respectively), which could be explained by the higher hydrophobicity of [Cho][Ol]. As expected, a higher molar fraction of [Cho][Ol] promoted increased toxicity against the studied bacteria. Authors from the same laboratory [52] also presented the synthesis and characterization of eight phosphonium and ammonium-based ILs (tetrabutylphosphonium phenylalanine ([P_4,4,4,4_][Phe]), tetrabutylphosphonium taurine ([P_4,4,4,4_][Tau]), tetrabutylphosphonium acetate ([P_4,4,4,4_][CH_3_COO]), tetrabutylammonium phenylalanine ([N_4,4,4,4_][Phe], tetrabutylammonium taurine ([N_4,4,4,4_][Tau]), tetrabutylammonium acetate ([N_4,4,4,4_][CH_3_COO]), tetrabutylphosphonium hydroxide ([P_4,4,4,4_][OH]) and tetrabutylammonium hydroxide ([N_4,4,4,4_][OH])) as well as their toxicity against six bacteria (*S. aureus*, *L. monocytogenes*, *Bacillus licheniformis*, *E. coli*, *Pseudomonas aeruginosa* and *A. hydrophilia*). The ILs, [N_4,4,4,4_][OH] and [P_4,4,4,4_][OH] were considered harmless against all bacteria. The other six ILs were considered practically harmless, and like in the studies presented by Weyin-Zerrer [53,54], no significant distinctions were observed in the toxicities between Gram-positive and -negative bacteria. In this study, the Gram-positive bacteria were also revealed to be more susceptible than Gram-negative bacteria due to the thicker and more lipophilic cell membranes of the Gram-positive. *B. licheniformis* exhibited more resistance than the other Gram-positive bacteria. The authors postulate that this could be due to its ability to form an endospore in response to an adverse environment. Comparing the EC_50_ and critical micellar concentrations (CMCs) values of [N_4,4,4,4_][OH] and [P_4,4,4,4_][OH], the CMCs values where lower than the EC_50,_ suggesting that these ILs tend to group together instead of interacting with the cell walls, leading to high EC_50_ values that correspond to the classification of harmless. For the other ILs studied, the CMCs values were higher than their EC_50_ values suggesting that the bacterial membranes promptly absorb the ILs molecules over micelle aggregation.

To better understand the mechanism by which ILs damage the cell membranes, Bhattacharya et al. [55] studied the antimicrobial activities of two ILs, 1-ethyl-3-methylimidazolium tetrafluoroborate ([C_2_mim][BF_4_]) and 1-butyl-3-methylimidazolium tetrafluoroborate ([C_4_mim][BF_4_]) against *E. coli* bacteria. Bacteria survival decreased with increasing IL concentration and, as expected, [C_4_mim][BF_4_] showed to be more toxic than [C_2_mim][BF_4_]. It was also observed a shape deforming effect of ILs on *E. coli,* especially when these bacteria were exposed to [C_4_mim][BF_4_]. The authors used a model of a lipid bilayer together with an X-ray reflectivity technique to determine that ([C_4_mim][BF_4_]) is more attuned to it, resulting in more disruption in the membrane, which the existence of negatively charged lipids can further strengthen. Since this study used simple models and biological membranes are much more complex, the authors suggested that more complex representations are needed to better understand the effect of ILs on cell membranes.

The role of ILs as antimicrobial agents in polymeric films for medical applications was studied recently [56]. This work evaluated the antimicrobial activity of 1-hexadecyl-3-methylimidazolium dimethyl-5-sulfoisophthalate ([C_16_mim][DMSIP]) and 1-octyloximethyl-3-methylimidazolium hexafluorophosphate ([C_8_OC_1_mim][PF_6_]) in a commercial polymer Pebax^®^Rnew, against five bacteria (*E. coli*, *Pseudomonas fluorescens (P. fluorescens)*, *Salmonella enterica (S. enterica)*, *L. monocytogenes* and *B. subtilis*). The films containing [C_16_mim][DMSIP]) (with concentrations of 1 and 5 wt %) showed good antimicrobial activity against *L. monocytogenes* and *P. fluorescens* while showing a poor effect against *E. coli* at 5 wt %. Films containing [C_8_OC_1_mim][PF_6_] displayed antimicrobial activity against *E. coli* at both concentrations and *P. fluorescens* only at 5 wt %. Although, none of the films/ILs displayed any antimicrobial activity against *S. enterica* and *B. subtilis*. Besides these results, the authors could observe that bacterial sensitivity is higher for ILs with long-chain lengths and suggested that these films could be used as antimicrobial films on furniture for sterile environments and as filters for aerating systems.

To determine and predict ILs toxicity, Weyhing-Zerrer et al. adopted a structure–activity relationship (SAR) applying defined structural motifs. In their first study [53], the toxicity of twenty-eight ILs on 12 g-positive and Gram-negative bacteria was analyzed. The ILs were selected to cover all SARs (cation alkyl side-chain effect, cation lipophilicity, cation–anion interactions and fluorinated anion stability). The selected bacteria were *L. monocytogenes*, *B. cereus*, *S. aureus*, *Enterococcus faecalis* (*E.* *faecalis*), *Lactobacillus sakei* (*L. sakei*), *Lactobacillus lactis* (*L. lactis*), *Salmonella typhimurium* (*S. typhimurium*), *E. coli*, *Citrobacter freundii* (*C. freundii*), *Proteus mirabilis* (*P. mirabilis*), *P. aeruginosa* and *V. fischeri*. For evaluating the cation alkyl side-chain effect, the ILs [C_n_mim][Cl] with n = 2, 4, 6, 8 and 10 were selected. As reported in other studies, the IL with a longer alkyl side-chain was the most toxic for all bacteria. The authors also observed that the previous assumption that Gram-negative bacteria were less susceptible to ILs than Gram-positive bacteria was not correct, and it is no longer valid to limit the differences in toxicity to their Gram-positive or negative classification. Regarding the cation lipophilicity effect on ILs toxicity, it was observed that for the three ILs tested (trimethyloctylammonium chloride [N_1,1,1,8_][Cl], dioctyldimethylammonium chloride [N_8,8,1,1_][Cl] and trioctylmethylammonium chloride [N_8,8,8,1_][Cl]), the Gram-positive bacteria were generally more susceptible than Gram-negative bacteria, apart from *V. fischeri*. For the analysis of the fluorinated anions, the following anions were selected: [CF_3_SO_3_], [BF_4_], [PF_6_] and tris(pentafluoroethyl)trifluorophosphate ([(C_2_F_5_)_3_PF_3_]). It was observed that MIC values of [C_8_mim][CF_3_SO_3_] did not differ from the ones obtained for [C_8_mim][Cl] for all bacteria. The influence of the [CF_3_SO_3_] anion was not visible, as previously observed by Mester et al. [42] for [C_n_mim]-based ILs with n ≥ 6, a measurable effect was detected on this anion was together with the [C_2_mim] cation. Due to its instability, the [BF_4_] anion is normally hydrolyzed in an aqueous solution, and the free fluorinated anions can potently inhibit Na^+^-K^+^-ATPase that can cause cell death. However, as observed with the [CF_3_SO_3_] anion, the MIC values were comparable to those of [C_8_mim][Cl]. For the [PF_6_] anion (a more stable anion), the same behavior was detected. Some unexpected behavior was observed with the [(C_2_F_5_)_3_PF_3_] anion. When paired with short-chain imidazolium cations, an increased toxic effect was detected and related to its more lipophilic nature. However, for the larger [C_6_mim], it resulted in more toxicity for some bacteria, no changes for others and no effects for bacteria where the combination with [C_2_mim] did exhibit toxicity. In fact, combining [N_1,1,1,8_][Cl] and trioctylmethylphosphonium ([P_8,8,8,1_]) with [Docusate] or [(CF_3_SO_2_)_2_N] shown decreased toxicity for Gram-negative bacteria than less hydrophobic anions. However, no explanation was found for this behavior. To better understand the effect of [(C_2_F_5_)_3_PF_3_] anion, the authors performed a more focused study [54], where they confirmed that Gram-positive bacteria were more susceptible to [(C_2_F_5_)_3_PF_3_] anion. *A. fischeri* was the most sensitive and susceptible to ILs from all the bacteria studied. It was also observed that even with a nontoxic cation, toxic effects could be observed with ([(C_2_F_5_)_3_PF_3_]) anion. Moreover, when combined with toxic cations, less or even nontoxic ILs could be obtained due to the reverse side-chain effect consequence of the more hydrophobic cation. The authors believe that the association of both bulky ions makes it so that the IL is no longer permeable through the cell membrane, thus barely inflicting any damage.

Other studies using a linear quantitative structure–activity relationship (QSAR) model that combines the ILs molecular structure with their microbial activity were presented by Ghanem and coworkers [57,58]. The first study [57] used twenty-five imidazolium-based ILs (C_n_, n = 4, 6, 8, 10 and 12) to evaluate the effect of the side-chain and the anion against four bacteria: *L.* monocytogenes, *S. aureus*, *E. coli* and *A. hydrophila*. As expected, the authors observed a notable decrease in EC_50_ values with the increase in cation side-chain length against all bacteria. In *A. hydrophila*, the toxicity increased 38 times from butyl (C_4_) to octyl (C_8_). The anion effect was analyzed using the 1-octyl-3-methylimdazolium ([C_8_mim]) cation and nine different anions, the toxicity decreased in the following order: [N(CN)_2_] > [Br] > [BF_4_] > [Cl] > [Asp] > [Gly] > [Ala] > [Pro] > [Ser] for all bacteria. A QSAR model was developed for each bacterium using multiple linear regressions. All the methods presented an *R*^2^ > 0.96, demonstrating that these models can be considered a reliable tool to determine the antimicrobial activity of the ILs against the studied bacteria. The second study [58] presented a QSAR model based on the functional group contribution method. In this work, four bacteria strains (*L. monocytogenes*, *S. aureus*, *E. coli* and *A. hydrophila*) and twenty-four ILs were used as well as data collected from previous studies and Hossain et al. [59]. The authors suggested that the toxicity of the ILs was related to their water solubility, which depends on their structure and not only on the number of carbons forming them. For example, imidazolium and pyridinium-based ILs (aromatic) have presented (not only in these studies but also in other studies previously reported) higher toxicities than piperidinium or pyrrolidinium-based ILs (non-aromatic). The EC_50_ values indicated the following toxicity trend: pyrrolidinium < piperidinium < pyridinium ≤ imidazolium. The use of a hydrophobic anion ([(CF_3_SO_2_)_2_N]) increased the ILs toxicity, confirming the influence of the hydrophobic anions on the toxic effect of ILs. The increase of the cation alkyl side-chain led to increased ILs hydrophobicity and consequently to a significant increase in their toxicity, as we have seen from the work of Weyhing-Zerrer and coworkers [53,54]. However, hydrophobicity does not tell the whole history. The model works for the ILs studied, but more research is needed with various ILs.

Recently, Abramenko and coworkers presented a review compiling the studies and developments of QSAR models for toxicity of ILs [60]. In this work, the authors summarize the degradation processes of ILs and the published computational models, indicating the data sets and databases used and sources. They also outline the factors influencing how harmful to living beings an IL can be and give an overview of current modeling techniques and descriptors to assess the toxicity of ILs and the mechanism in place.

### 2.2. Fungi

Besides bacteria, other microorganisms were studied to determine the microtoxicity of ILs, like *Candida albicans* (*C. albicans*), *Fusarium* sp. or *Aspergillus fumigatus* (*A. fumigatus*). However, there are only a few studies dedicated only to the toxicity of ILs towards fungi. Most of the authors prefer to perform studies more complete, studying the toxicity of ILs against different microorganisms (bacteria and fungi).

**Figure 4:**
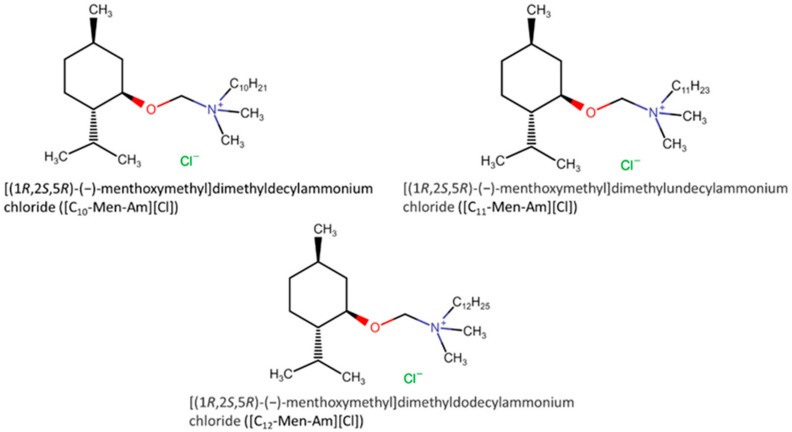
Structures of alkyl[(1R,2S,5R)-(−)-menthoxymethyl] dimethylammonium chloride [61].

The continuous search for new ILs with specific properties (for example, low viscosity) and low toxicity directed some researchers [61] to use natural components in the ILs synthesis. DL-menthol is one of the natural products studied as a natural base for new solvents and ionic liquids. (1R,2S,5R)-(−)-menthol was used to produce [(1R,2S,5R)-(−)-menthoxymethyl] dimethylalkylammonium chloride ([C_n_-Men-Am][Cl]) (n = 10, 11, 12) ILs (see Figure 4) and their antifungal activities were studied toward two strains of *C. albicans*. Regarding the SC5314 strain, no correspondence was observed between the antimicrobial activity and the alkyl chain length of ILs, although the DSY1050 strain presented a higher vulnerability towards the [C_10_-Men-Am][Cl] (MIC100 = 5 μM) and [C_11_-Men-Am][Cl] (MIC100 = 5 μM). Contrarily, the minimal fungicidal concentrations (MFC) were higher against the DSY1050 strain than the SC5314 strain for all ILs. It is known that *C. albicans* can generate a biolayer comprised of cells with a modified metabolism and enveloped by an extracellular matrix, making it resistant to antifungals. The presence of these ILs causes a 20–30% detachment of the cells from the surface in all concentrations and ILs, except for [C_11_-Men-Am][Cl] at 100 μM, with 40%. It was also observed that these ILs inhibited the filamentation of this microorganism at an IL concentration of 10 μM, by various 75–89.5% and due to their low hemolytic activity, all the synthesized 1R,2S,5R)-(-)-menthol-based ILs can be considered disinfectants.

Following the same idea of using natural compounds in the ILs synthesis, Bromberger and coworkers [62] recently incorporated pyrithione (2-mercaptopyridine N-oxide), a derivate of naturally occurring aspergillic acid in ILs. This compound is an efficacious antimicrobial, but it can only stop the growth of bacteria and ineffective against viruses. The activity of twenty pyrithione-based ILs against eleven bacteria strains, two yeast strains and three viruses was presented. This study revealed that the pyrithione-based ILs keep the high antimicrobial activity that characterizes the pyrithione, with MIC values between 1 and 30 mg·L^−1^ against all the bacterial strains, being confirmed by the diffusion tests with *Geobacillus stearothermophilus* (*G. stearothermophilus*). However, the trioctylmethylammonium pyrithione ([N_8,8,8,1_][Pyr]) was an exception, presenting unusually small inhibition zones in *E. coli* and *G. stearothermophilus* diffusion tests. The authors suggested that a probable cause for this behavior was the restricted movement of the cation in the medium, likewise impeding that of the pyrithione anion. It was also observed that these ILs have a predominant bacteriostatic effect on Gram-negative bacteria, only inhibiting the bacterial growth without causing its dead, limiting applying these ILs as a biocide. This behavior was not observed in the Gram-positive bacterial strains. The toxicity tests on yeast were performed towards *Saccharomyces cerevisiae* (*S. cerevisiae*) and *Candida tropicalis* (*C. tropicalis*). The results showed that pyrithione-based ILs maintain their antifungal activities in IL form and the most effective ones were trimethylhexadecylammonium pyrithione ([N_1,1,1,16_][Pyr]) with a MIC-= 2.3 mg·L^−1^ and [N_8,8,8,1_][Pyr] with a MIC = 3.7 mg·L^−1^. The results obtained in this study revealed the promising antimicrobial applications of these ILs and their effectiveness against viruses. However, the authors suggest that further research is needed.

The same laboratory that previously reported the toxicity of L-phenylalanine-derived ILs [35] towards *A. fischeri* also analyzed the response of other bacteria and fungi [63]. For the bacteria, the Gram-positive were more susceptible than the Gram-negative and like in the previous study, the alkyl side-chain effect was observed. In the case of the L-phenylalanine-derived ILs with a pyridinium base, for the Gram-positive bacteria, the MIC values, for 48 h of exposure, decreased from C_2_ to C_10_, kept equal between C_10_ and C_12_ and then increased until C_16_. However, the values at C_14_ and C_16_ were lower than the obtained for C_8_. In the case of the Gram-negative bacteria, the MIC values decreased from C_2_ to C_10_ and then increased until C_16_ with C_14_ and C_16_ above the same limit of that of C_8_. However, for imidazolium and cholinium-based ILs, the Gram-negative bacteria MIC values decreased until C_12_. The values for C_14_ and C_16_ were much higher than the ones obtained for C_8_. The antifungal results were similar for all the cation structures, where the MIC values decrease from C_2_ to C_14_. At the same time, at C_16_, depending on the base (pyridinium, imidazolium and cholinium) and the fungal strain, the MIC can remain constant, decrease or increase, except for the *Candida lusitaniae* (*C. lusitaniae*), where the C_16_ value is higher than C_8_. The authors also studied the biodegradation effects of the C_10_ ILs (due to the occurring of the cutoff effect) where the imidazolium-based showed 44% of biodegradation, followed by pyridinium (36%) and cholinium (27%); none of them can be considered biodegradable.

Jordan et al. [64] designed and developed a green synthesis of a series of amino acid-based ILs and performed toxicity and biodegradation studies of these ILs against eight bacteria and twelve fungal strains. Regarding the antimicrobial activity, all the ILs and neutral derivatives had low antimicrobial activity except one of the neutral L-phenylalanine IL analog, the ethyl(2-(((S)-1-ethoxy-1-oxo-3-phenylpropan-2-yl)amino)-2-oxoethyl)-L-prolinate. This compound had the highest toxicity values with IC_95_ = 125 and 250 μM against *S. aureus* and *S. epidermidis* (both Gram-positive bacteria). The results showed that all the ILs and neutral derivatives did not have high antifungal activity, even the IL, (S)-4-(Dimethylamino)-1-(2-((1-ethoxy-1-oxo-3-phenylpropan-2-yl)amino)-2-oxoethyl)pyridin-1-ium bromide, that had the lowest values (IC_95_ > 500 μM) against all fungal strains. The biodegradability tests of these ILs showed that the IL, (S)-1-(2-((1-ethoxy-1-oxo-3-phenylpropan-2-yl)amino)-2-oxoethyl)pyridin-1-ium bromide had the highest biodegradation (63%), but it could not be considered readily biodegradable since it took more than 10 days after degradation reaches 10% theoretical oxygen demand (ThOD)—corresponding to the total amount of oxygen required for a chemical to complete its oxidation process) in accordance with Organization for Economic Cooperation and Development (OECD) guidelines [65]. The addition of an ether group at the 3-position—(S)-1-(2-((1-ethoxy-1-oxo-3-phenylpropan-2-yl)amino)-2-oxoethyl)-3-methoxypyridin-1-ium bromide—induced decreased the biodegradation to 52%. The lowest biodegradation value was obtained by the (S)-4-(2-((1-ethoxy-1-oxo-3-phenylpropan-2-yl)amino)-2-oxoethyl)-4-methylmorpholin-4-ium bromide (17%). The presence of the OH group in tyrosine-based ILs did not cause increased biodegradation compared to phenylalanine-based ILs. The authors preferred green IL was the (S)-1-(2-((1-Ethoxy-1-oxo-3-phenylpropan-2-yl)amino)-2-oxoethyl)pyridin-1-ium bromide due to its efficient and relatively short synthesis, low antimicrobial activity and even though not being possible to consider it readily biodegradable, it presented good biodegradation properties compared with all the others ILs synthesized.

**Figure 5:**
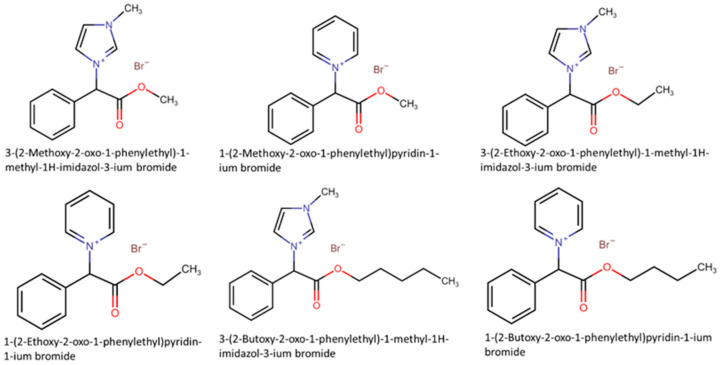

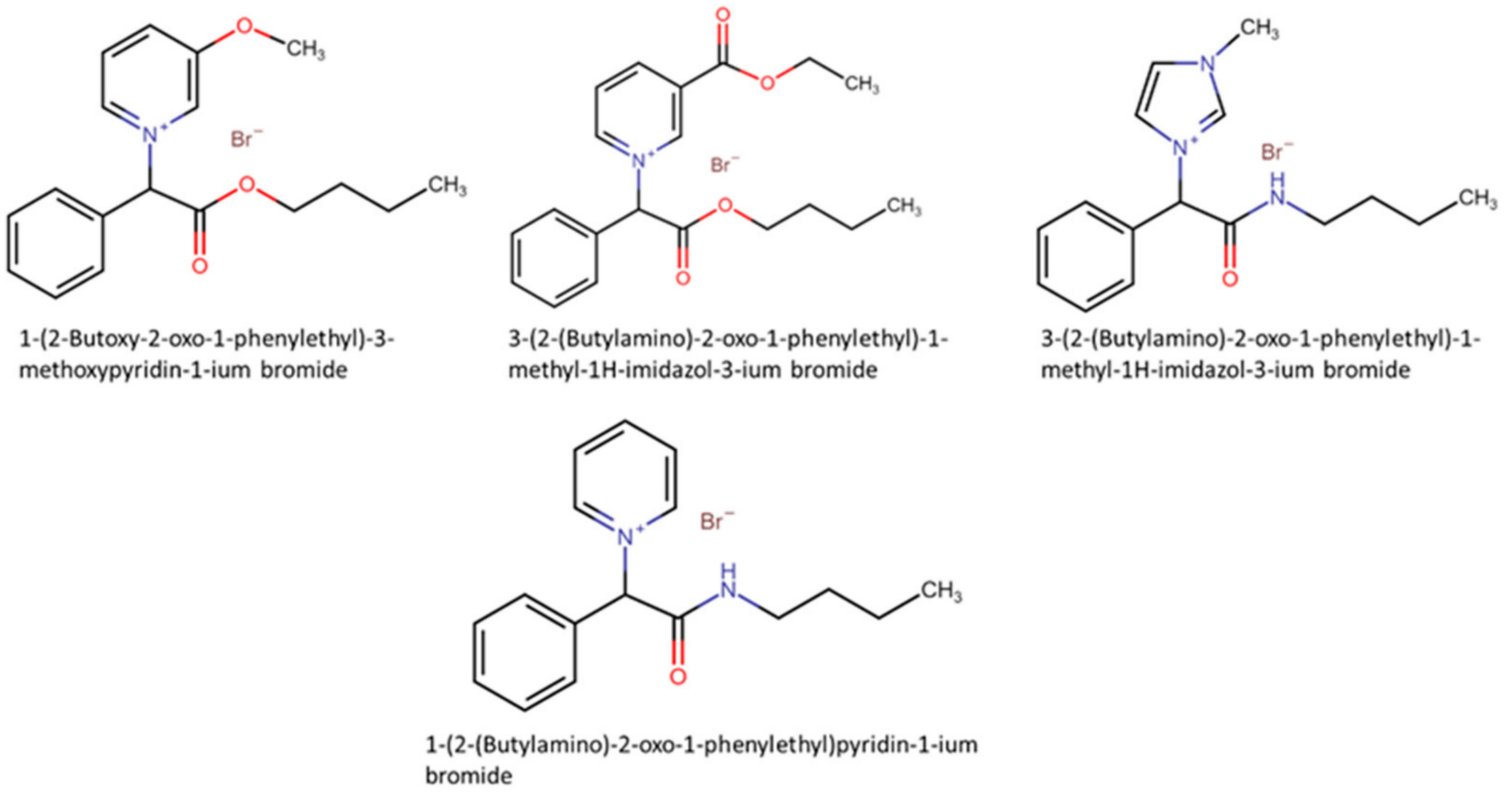
Structures of alkyl[(1R,2S,5R)-(−)-menthoxymethyl] dimethylammonium chloride [66].

Mandelic acid is an aromatic acid found in small quantities in plant tissues and can also be easily converted from L-phenylalanine and phenyl acetic acid by a wide range of organisms. To obtain biodegradable ILs, Prydderch et al. [66] synthesized 10 mandelic acid ILs (Figure 5) and evaluated their toxicity and biodegradability. The toxicity studies of mandelic acid ILs were performed against twelve fungal strains (four ATTC strains and eight clinical isolates of fungi—five yeasts and three filamentous fungi) and eight bacterial strains (three ATTC strains and five clinical isolates). Against fungi, all ILs, but one showed no effect (IC_50_ for yeasts and IC_80_ for filamentous fungi) up to their corresponding maximum concentration (1.0 mM or 2.0 mM depending on the IL). Only 3-(2-ethoxy-2-oxo-1-phenylethyl)-1-methyl-1H-imidazol-3-ium bromide displayed toxic effects towards both strains of *Candida krusei* (*C. krusei*) used in the study, even though analog ILs with longer cation side-chain (3-(2-butoxy-2-oxo-1-phenylethyl)-1-methyl-1H-imidazol-3-ium bromide and 1-(2-butoxy-2-oxo-1-phenylethyl)pyridin-1-ium bromide) were also tested. Furthermore, interesting is the fact that the 1-methylimidazolium and pyridinium n-butyl ester ILs inhibited *C. Tropicalis* at 2 mM at the 24 h mark but showed no inhibition after the 48 h period, suggesting that it overcame the initial suppression. The toxicity study against bacterial strains revealed that all the ILs did not have high toxicity (IC_95_) against the bacterial strains except for 3-(2-Ethoxy-2-oxo-1-phenylethyl)-1-methyl-1H-imidazol-3-ium bromide toward *S. aureus* (both strains), *S. epidermidis* and *Enterococcus* sp., and 1-(2-butoxy-2-oxo-1-phenylethyl)pyridin-1-ium bromide against *S. aureus* ATTC 6538 and *S. epidermidis*. A further antibacterial screening against one Gram-positive bacterial strain (*B. subtilis*) and four Gram-negative bacteria (*E. coli*, *P. fluorescens*, *P. putida* CP1, and *P. putida* KT 2440) showed that the highest levels of inhibition were obtained by: 3-(2-Butoxy-2-oxo-1-phenylethyl)-1-methyl-1H-imidazol-3-ium bromide against *E. coli* (IC_50_ = 6.25–12.5 mM) and *P. putida* CP1 (IC_50_ = 6.25–12.5 mM), 3-(2-(Butylamino)-2-oxo-1-phenylethyl)-1-methyl-1H-imidazol-3-ium bromide against *E. coli* (IC_50_ = 6.25–12.5 mM) and 1-(2-(Butylamino)-2-oxo-1-phenylethyl)pyridin-1-ium bromide against *E. coli* (IC_50_ = 6.25–12.5 mM) and *P. fluorescens* (IC_50_ = 6.25–12.5 mM). The cation alkyl side-chain effect was also observed, and toxicity increases according to methyl ester < ethyl ester < *n*-butyl ester/amide, being clear that the microbial toxicity was influenced by the lipophilicity of the IL structures. Regarding the biodegradation tests, none of the ILs studied obtained more than 60% of biodegradation after 28 days and could not be considered readily biodegradable. The 3-(2-(butylamino)-2-oxo-1-phenylethyl)-1-methyl-1H-imidazol-3-ium bromide and 1-(2-(butylamino)-2-oxo-1-phenylethyl)pyridin-1-ium bromide did not biodegrade at all.

Thamke and coworkers [67] presented the toxicity of five ILs: 1-butyl-3-methylimidazolium bromide ([C_4_mim][Br]), trihexyltetradecylphosphonium dicyanamide ([P_6,6,6,14_][N(CN)_2_]), 1-decyl-3-methyl imidazolium tetrafluoroborate ([C_10_mim][BF_4_]), benzyldimethyltetradecylammonium chloride ([BTDA][Cl]) and 1-butyl-4-methylpyridinium chloride ([C_4_mpy][Cl]) against bacteria (Gram-positive: *Bacillus subtilis*, *S. aureus*; Gram-negative: *E. coli*, *P. aeruginosa*, *Bradyrhizobium japonicum* (*B. japonicum*), *Thiobacillus novellus* (*T. novellus*), and *Alcaligenes faecalis* (*A. faecalis*), these last three are abundant in agricultural soil), fungi (*C. Albicans* and *Aspergillus brasiliensis (A. brasiliensis)*), plants (*Triticum aestivum* seeds and onion bulbs (*Allium cepa*) and animal cells. The toxicology tests in bacteria revealed that [BTDA][Cl] was very active against all bacteria, and the pyridinium-based [C_4_mpy][Cl] showed to be more toxic than the imidazolium-based IL ([C_4_mim][Br]) for both Gram-positive and negative bacteria. In the case of agronomic soil bacteria, the *Bradyrhizobium japonicum* was more sensitive against all ILs. The fungi tests showed that both fungi were more resistant than bacteria towards the ILs studied. For both fungi, [C_4_mim][Br] and [C_4_mpy][Cl] did not display growth inhibition, even at a concentration of 1000 μg. *C. albicans* was less resistant than *A. brasiliensis* to [P_6,6,6,14_][N(CN)_2_] (10 μg and 1000 μg, respectively), [C_10_mim][BF_4_] (10 μg and 500 μg, respectively) and [BTDA][Cl] (10 μg and 100 μg, respectively).

ILs can be divided into two groups considering their chemical behavior: aprotic ionic liquids (APILs) and protic ionic liquids (PILs). AILs have been widely used, and the more common are the imidazolium and pyridinium-based ILs. PILs are synthesized through proton transfer from a Brønsted acid to a Brønsted base. Recently, PILs have gained attention from the academic community due to their simple synthesis, low-cost purification, and alleged biodegradability [68,69,70]. Oliveira et al. [71] studied not only the toxicity (Microtox and antimicrobial tests), but also the biodegradability of four PILs (N-methyl-2-hydroxyethylammonium acetate [OHC_2_C_1_NH_2_][CH_3_COO], N-methyl-2-hydroxyethylammonium propionate [OHC_2_C_1_NH_2_][C_2_H_5_COO], N-methyl-2-hydroxyethylammonium butyrate [OHC_2_C_1_NH_2_][C_3_H_7_COO] and N-methyl-2-hydroxyethylammonium pentanoate [OHC_2_C_1_NH_2_][C_4_H_9_COO]). The antimicrobial activity test was performed on bacteria *E. coli* and *S. aureus*, on the mold *Fusarium* sp. and on the yeast, *C. albicans.* The toxicity was performed on *A. fischeri*. Regarding the antimicrobial activity, this study suggested that all PILs had activity against the microorganisms, mainly for the mold and yeast. An increase in alkyl chain length was accompanied by increased toxicity for the bacteria tested until the “cutoff” at the butyl chain. This effect was more notorious on *E. coli* than *S. aureus*. The toxicity tests of PILs towards *A. fischeri* followed the tendency: [OHC_2_C_1_NH_2_][CH_3_COO] (EC_50_ = 900.63 mg·L^−1^; EC_50_ = 962.54 mg·L^−1^) < [OHC_2_C_1_NH_2_][C_2_H_5_COO] (EC_50_ = 620.78 mg·L^−1^; EC_50_ = 887.81 mg·L^−1^) < [OHC_2_C_1_NH_2_][C_3_H_7_COO] (EC_50_ = 591.33 mg·L^−1^; EC_50_ = 717.00 mg·L^−1^) < [OHC_2_C_1_NH_2_][C_4_H_9_COO] (EC_50_ = 456.62 mg·L^−1^; EC_50_=551.61 mg·L^−1^) for 5 and 15 min of exposure. The biodegradation assays revealed that all the PILs have a nonbiological treatable nature since the ratio between the biochemical oxygen demand (BOD) and chemical oxygen demand (COD) was lower than 0.3.

**Figure 6:**
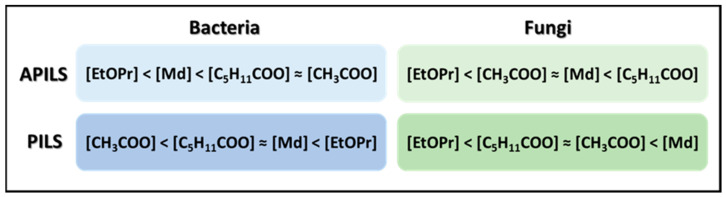
The anion effect on PILs and APILs against bacteria and fungi [72].

To better understand how the variation in ionic structure of ILs will influence their microbial activities, Reid and coworkers [72] prepared twelve *N,N*,*N*-trimethylethanolammonium (cholinium)-based APILs: [Cho], *N,N,N*-trimethyl-2-(2-hydroxyethoxy)ethylammonium [TMHEEA], *N,N,N*-trimethyl-2-(2-ethoxyethoxy)ethylammonium [TMEEEA] and twelve N,N-dimethylethanolammonium-based PILs: *N,N*-dimethylethanolammonium [DMEtAH], *N,N*-Dimethyl-2-(2-hydroxyethoxy)ethylammonium [DMHEEAH], *N,N*-Dimethyl-2-(2-ethoxyethoxy)ethylammonium [DMEEEAH], with the anions: acetate [CH_3_COO], hexanoate [C_5_H_11_COO], mandelate [Md] and 3-ethoxypropionate [EtOPr]. The antimicrobial activities of these ILs were tested against twelve fungal strains: *C. albicans* ATCC 44859, *C. albicans* ATCC 44859, *Candida parapsilosis* (*C. parapsilosis*) ATCC 22019*, C. krusei* ATCC 6258, *C. krusei* E28, *C. tropicalis* 156, *Candida glabrata* (*C. glabrata*) 20/I, *Candida lusitaniae* (*C. lusitaniae*) 2446/I, *Trichosporon asahii* (*T. asahii*) 1188, *A. fumigatus* 231, *Absidia corymbifera* (*A. corymbifera*) 272, *Trichophyton mentagrophytes* (*T. mentagrophytes*) 4459 and eight bacterial strains (*S. aureus* ATTC 6538, *S. aureus* MRSA HK5996/08, S*. epidermidis* HK6966/08, *Enterococcus* sp. HK14365/08, *E. coli* ATTC 8739, *K. pneumoniae* HK11750/08, *K. pneumoniae* ESBL HK14368/08 and *P. aeruginosa* ATTC 9027). From the twenty-four ILs studied, only eight presented MIC (IC_95_) values greater than 2000 μM against all the microorganisms’ strains analyzed. However, the authors mention that although the results obtained do not classify these ILs as nontoxic, they do not have acute microtoxicity. Regarding the antimicrobial activity, most of the ILs presented MIC values higher than 2000 μM, and *S. epidermidis* (Gram-positive bacteria) was the most susceptible bacteria. The [TMEEEA] and ([DMEEEAH])-based ILs were more toxic towards bacteria strains, and comparing the cation influence, the [DMEEEAH]-based ILs exhibited higher toxicity than [TMEEEA]-based ILs. The anion influence was different for each class of ILs (Figure 6), in APILs the trend of toxicity followed [EtOPr] < [Md] < [C_5_H_11_COO] ≈[CH_3_COO], while the PILs toxicity trend was [CH_3_COO] < [C_5_H_11_COO] ≈[Md] < [EtOPr]. As reported in other studies, the ILs toxicities were higher towards Gram-positive bacteria (*S. aureus*, *S. epidermidis* and *Enterococcus* sp.) than towards Gram-negative bacteria (*E. coli*, *K. pneumoniae* and *P. aeruginosa*). The authors point out that the responsiveness of *S. aureus* to all the ILs tested, with a wide range of IC_95_ values, could be used to assess the antibacterial properties of these ILs. However, they also warn us of the perils of using a single bacterial strain to represent the antimicrobial activity of an IL. The antifungal activity screening revealed that the MIC values were higher for fungi than bacteria. For [Cho][CH_3_COO] and [DMEtAH][EtOPr], the MIC values obtained were higher than 2000 μM for all fungal strains and can be considered nontoxic to these strains. The [DMHEEAH]-based ILs were toxic (MIC < 500 μM) or moderate toxic (MIC > 500–< 2000 μM) for all fungal strains, while the corresponding APILs ([TMEEEA]) presented greater MIC values and could be considered moderately toxic for *Candida parapsilosis*, *C. krusei* and *C. tropicalis* (except for [TMEEEA][C_5_H_11_COO] that was toxic against *C. krusei*) and nontoxic for the other fungal strains. Different anions differently affect the fungal toxicities of each IL class, although not in a consistent manner as observed in the bacteria. For PILs, the trend of increasing fungal toxicity follows [EtOPr] < [C_5_H_11_COO] ≈ [CH_3_COO] < [Md], while AILs trend as [EtOPr] < [CH_3_COO] ≈ [Md] < [C_5_H_11_COO]. It was observed that ILs with longer ether chain functional groups in the cation presented higher antimicrobial activities. Small increases in toxicity between anion structures could be attributed to their more lipophilic nature. The APILS containing cations with the 2-(2-ethoxyethoxy)ethyl functional group presented higher MIC values for all bacteria and fungi studied compared to their protic analogs. Based on the toxicity results, the authors suggest that low MIC values for the [TMEEEA] and [DMEEEAH]-based ILs could result from a slow process where the functional ether chain turns into a shorter functional chain with a hydroxy terminal group. The other ILs exhibited higher MIC values and can be considered biodegradable. Summing up, the ILs that exhibited the lowest toxicity (MIC values higher than 2000 μM) to all the twenty microbial strains analyzed were [Cho][C_5_H_11_COO], [Cho][Md], [Cho][EtOPr], [TMHEEA][Md], [TMHEEA][EtOPr], [DMEtAH][CH_3_COO], [DMEtAH][C_5_H_11_COO] and [DMEtAH][Md].

The influence of six trihexy(tetradecyl)phosphonium-based ILs on the structure of soil microbial community (more properly in urban park microcosms) was presented by Sydow et al. [73]. The ILs studied were: trihexytetradecylphosphonium bromide ([P_6,6,6,14_][Br]), trihexytetradecylphosphonium bis(2,4,4-trimethylpentyl)phosphinate ([P_6,6,6,14_][TMPP]), trihexytetradecylphosphonium 3-amino-1,2,4-triazolate ([P_6,6,6,14_][3AT]), trihexytetradecylphosphonium benzotriazolate ([P_6,6,6,14_][Bt]), trihexytetradecylphosphonium bis(trifluoromethylsulfonyl)imide ([P_6,6,6,14_][(CF_3_SO_2_)_2_N]) and trihexytetradecylphosphonium (dicyano)imide ([P_6,6,6,14_][N(CN)_2_]). Soil composition was presented in previous work [74] and is mainly composed of clay (≈4%), silt (≈83%) and sand (≈13%) and the main characteristics were organic carbon (5.44 ± 0.31 g·kg^−1^), nitrogen (0.57 ± 0.07 g·kg^−1^), phosphorous (0.080 ± 0.005 g·kg^−1^) and pH ≈ 6.95. Regarding biodegradation, the results showed that after the incubation time (100 days), both cation and anion were still detected in all the samples, where the amount of cation was influenced by the type of anion. The smallest amount of cation was observed in the presence of [Br] anion (9%), and the highest was obtained with the anion [N(CN)_2_] (60%). However, the [Br] anion was not detected, and the order of residual anions was: [N(CN)_2_] (46%), [Bt] (41%), [3AT] (38%), [(CF_3_SO_2_)_2_N] (26%) and [TMPP] (12%). The study of the evolution of CO_2_ in the microcosms revealed that ILs did not significantly inhibit the respiration activity of soil microbiota, although the soil samples containing [P_6,6,6,14_][Br] presented higher CO_2_ evolution curves. After exposure to sublethal concentrations of these ILs, the structure of the soil microbial community was significantly influenced by ILs presence. For example, in soils treated with [P_6,6,6,14_][(CF_3_SO_2_)_2_N], the contribution of *Alphaproteobacteria* and *Gammaproteobacteria* increased by 24 and 7%, respectively. The *Bacilli* class increased almost 10%, in soils treated with [P_6,6,6,14_][TMPP] and [P_6,6,6,14_][(CF_3_SO_2_)_2_N]. However, the contribution of *actinobacteria* and *Planctomycetia* class decreased between 1 to 7% in all samples. In conclusion, introducing phosphonium-based ILs into soils at sublethal concentrations may result not only in decreased biodiversity due to toxic effects but also in an increased population of IL-degrading bacteria.

**Figure 7:**
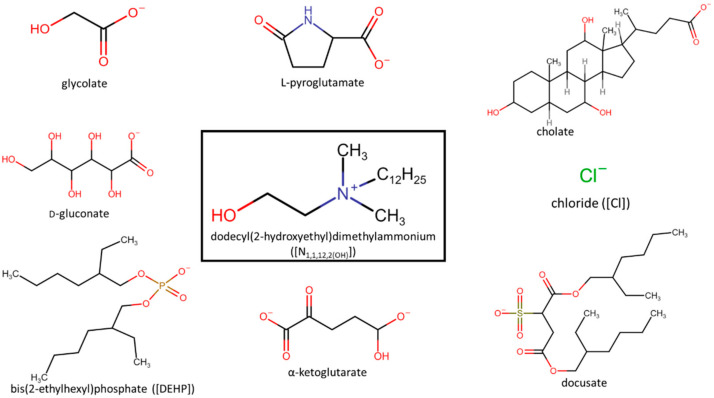
Ionic liquids studied by Kaczmarek et al. [75].

The search for new biocompatible adjuvants with low toxicity for use in pesticides formulation prompted Kaczmarek and coworkers [75] to evaluate the microbial toxicity of eight dodecyl(2-hydroxyethyl)dimethylammonium ([N_1,1,12,2(OH)_])-based ILs (Figure 7) and two ammonium salts (didecyldimethylammonium chloride [N_10,10,1,1_][Cl] and alkyl-benzyldimethylammonium chloride [BA][Cl]) against ten bacteria and two yeasts.

The bacteria used in this study were *S. aureus*, *S. epidermidis*, *E. faecalis*, *B. subtilis*, *Micrococcus luteus* (*M. luteus*), *E. coli*, *P. aeruginosa*, *Serrata marcescens* (*S. marcescens*), *Proteus vulgaris* (*P. vulgaris*) and *Moraxella catarrhalis* (*M. catarrahalis*), and the yeasts used were *C. albicans* and *Rhodoturula rubra*. The results showed that the Gram-positive bacteria were more susceptible to ILs toxicity than the Gram-negative bacteria and yeasts. *S. epidermis* was the more resistant from the Gram-positive bacteria studied, while *S. Marcescens* was the more susceptible strain from the Gram-negative bacteria. Regarding yeasts, *C. albicans* was more susceptible than the *R. rubra* to ILs toxicity. From all the ILs studied, [N_1,1,12,2(OH)_][docusate] was the least toxic against all organisms. In this work, it was also observed that the chemical structures of the ILs have a huge impact on their toxicity against these organisms. The ILs with the glycolate, α-ketoglutarate and L-pyroglutamate anions were more harmful to bacteria, while the ILs with the anions glycolate and bis(2-ethylhexyl)phosphate showed more toxicity against both species of fungi. The [N_1,1,12,2(OH)_][Cl] showed the same or higher toxicity of all ILs, except the [N_1,1,12,2(OH)_][docusate], against most the microorganisms (see Table 2). The commercial salts exhibited higher toxicities than the synthesized ILs. Based on the natural origin of the anions, the biodegradability of the cation and ready biodegradability of the synthesized anions, the authors assumed that these ILs should be characterized by at least moderate susceptibility to microbial degradation. It is important to mention that the ILs with natural anions displayed significant toxic effects against the microorganisms studied. However, the selected anions (nonamphiphilic anions) did not enhance the biological effectiveness and were weaker adjuvants when compared to Biopower (commercial adjuvant produced by Bayer CropScience).

Ionic liquids have been widely studied for biomass fractionation processes and even in treating paper artifacts, paper restoration and antifungal agents for wood preservation [76,77,78,79,80]. The ILs that qualify for this application presents several characteristics like the presence of bulkier substituents, halogens or oxygen heteroatoms in the cation side-chain or even the presence of sp^2^ double bonds (like alkyl radicals) that promote the antifungal character of ILs due to a better interaction with the fungal cell membrane. This character was observed by Dimitric et al. [77] when studying the ability of 1-amino-2-propanol-based PILs as potential fungi and bacteria removers from paper artifacts, where all the ILs synthesized presented antimicrobial activity. The PILs containing mandelate and trifluoroacetate anions had the lowest antimicrobial activity. In contrast, the ILs with chloroacetate, 3-chloropropinate and 4-chlorobutyrate anions had the highest antibacterial and antifungal activity, even at the lowest concentrations being a good alternative to conventional fungicides for paper cleaning and conservation. The substituents’ positions in heterocyclic cations can also contribute to the antifungal properties of ILs and the type of anion. The antifungal activity of 1-butyl-3-methylimidazolium acetate [C_4_mim][CH_3_COO] (an IL commonly studied for paper reinforcement processes with similar structural characteristics with popular antifungal agents like miconazole or clotrimazole) towards *Chaetomium globosum* (*C. globosum*) was 90% higher than 1-butyl-3-methylimidazolium chloride [C_4_mim][Cl][79]. In the ammonium-based ILs, the anion influence appears to be more pronounced on their antifungal character, like in the case of dimethyl-dodecyl-methoxy hexylammonium formate that has a 100% increase in the ED_100_ (preservative concentrations retarding the fungal growth rate by 100% than the reference) values towards *C. globosum* compared to the dimethyl-dodecyl-methoxy hexylammonium acetate. In didecyldimethylammonium-based ILs, the increasing toxicity efficiency against *Coniophora puteana* follows nitrate << 3-aminotriazolate < 4-chloro-2-methylphenoxyacetate ≈nitrite. Although for the pyridinium-based ILs, the anion presents a lower influence on ILs antifungal activity [78,80].

QSAR models can also be applied in the prediction of antifungal activities of ILs. Cho et al. [81] developed six QSAR models using linear free energy relationship (LFER) descriptors to predict MIC and MBC of ILs toward *E. coli, S. aureus* and *C. albicans*. The models developed presented acceptable results. The predictions models of log 1/MIC and log 1/MBC to *E. coli* obtained an *R*^2^ = 0.900 with standard error (SE) = 0.430 and an *R*^2^ = 0.934 with SE = 0.490, respectively (SE always in log unit of μM). For *S. aureus*, the models developed obtain an *R*^2^ = 0.910 with SE = 0.490 for log 1/MIC and an *R*^2^ = 0.947 with SE = 0.350. In the yeast, *C. albicans*, the log 1/MIC predictive model had an *R*^2^= 0.892 with SE = 0.362, and for the log, 1/MBC an *R*^2^ = 0.947 with SE = 0.350. In a second study [82], the same authors develop a new QSAR model where they used a bigger set of experimental toxicity data, including data for ILs toxicity against bacteria, fungi, enzymes and cell lines. In this work, the model developed (also using LFER predictors) presented an *R*^2^ = 0.901 with an SE of 0.426. Instead of using LFER descriptors, Yan and coworkers [83] decided to apply matrix norm and topological indexes to develop two new QSAR models to predict the antifungal activity of ILs against *C. albicans*. A total of 141 MIC and 85 MBC experimental data of ILs against *C. albicans* were collected. The models developed presented an *R*^2^ = 0.930 with a SE of 0.254 for pMIC (predictive MIC) and an *R*^2^ = 0.873 and a SE of 0.243. These models presented similar square correlation coefficients to the ones developed by Cho et al. [81], but smaller standard error, showing that these two-matrix norm index-based models are also reliable to determine MIC and MBC of ILs against *C. albicans*. All these models could be a faster, helpful and reliable supplement method to the experimental toxicity tests.

In conclusion, the type of cation and the cation side-chain length are the most responsible for the toxicity of ILs on microorganisms. In general, Gram-positive bacteria are more susceptible to ILs toxicity than Gram-negative bacteria, probably due to their thick cell membrane, although more studies are needed to better understand how ILs interact with the bacteria cell membrane. Most of the ILs studied had antimicrobial activity. They could be used as antibacterial or antifungal disinfectants, although the biodegradation studies reveal that most of the ILs could not be considered biodegradable or readily biodegradable, even the ones produced from natural compounds.

## 3. Effect of Ionic Liquids on Plants

Pawlowska et al. [21] recently presented a review where the cytotoxic information of 319 ionic liquids was collected and analyzed. According to the authors, most of the studies indicate that the type and nature of the cation, the length of alkyl chains in the substituent, the type of anion and cation–anion interactions are important. In addition, it is important to determine which plants are affected by the tested compounds and the environment in which the research is conducted. The correlation between the toxicology of the tested ILs and the time of exposure of plants to them is also a matter to consider in the many studies analyzed in this review. Besides the existing toxicology studies, there is still a poor understanding of the underlying processes that harm plants.

It is cross-sectional to all articles that studying the toxicology of ionic liquids in numerous plants, like algae, cereals (barley, wheat, maize and rice), vegetables, Pine trees, Eucalyptus and others gives evidence that their growth is inhibited by increasing the concentration of ionic liquid. Huijun Liu et al. [84] and Tong Liu et al. [85] confirmed these facts in studies with rice seedlings and *Vicia faba* seedlings where the increase in IL concentration decreased the stem and root size and reduced the number of lateral roots and root hairs. However, Huijun Liu et al. [86] and DingDong Liu et al. [87] described that some level of adaptation occurs when the seedlings have enough time thanks to low concentrations.

Toxicology studies have shown that the length of the cation alkyl chain influences the toxicity of ILs, since it increases their lipophilic properties and, as we have already seen at this point, facilitating the interaction with the cell membrane, making it less rigid and wavier. Huijun Liu et al. [88] by studying the effect of chiral ILs (1-alkyl-3-methyl imidazolium tartrate) on *Scenedesmus obliquus* (*S. obliquus*) and Yu et al. [89] through studying the toxicity of eight common ILs ([C_4_mim][CH_3_COO], [C_4_mim][C_7_H_5_O_2_], [C_4_mim][BF_4_], [C_4_mim][Br], [C_12_mim][Br], [C_4_py][Br], [C_8_mim][Br], [C_4_mim][Br] and [C_2_mim][Br]) on wheat seedlings, found that the increase of ILs lipophilicity is responsible for the increase of the membrane permeability to ILs as it comes into contact with proteins, facilitating its passage into cells, increasing the toxicity of ILs. With the intrusion of ILs into the cell, various constituents undergo several mutations and behavioral changes that usually lead to cell death, as proven by Xia et al. [90], when they studied the effect of four ILs ([C_6_mim][NO_3_], [C_6_mim][Cl], [C_6_mpy][Cl] and [C_6_mpy][Br]) on photosystem and cell structure of *S. obliquus*. The authors found out that besides affecting the cell membrane permeability, the ILs also damaged the photosynthetic system II (PSII), consequently inhibiting the primary reaction of photosynthesis. The type of cation and anion is also a factor to consider. In this case, the imidazolium ILs affect more this type of algae than the pyridinium ILs and analyzing the anion. The chloride anion showed a lower effect than the nitrate or bromide anions. Several authors also described the effect on photosynthesis since the increase of the cell membrane permeability allows ILs to enter the cell-damaging the thylakoids in the chloroplast and block the chlorophyll production, and avoid the electrons transfer from PSII to the photosynthetic system I (PSI) [91]. When the plant enters into oxidative stress due to high ROS values, SOD and CAT activities increase. Therefore, in most studies, it is not surprising that the activities of these enzymes, when the cell is exposed to IL, will be higher than the control cells. However, the activity of these two antioxidants decreases when IL concentrations exceed the IC_50_ value [87,88,91,92,93,94,95]. Fan and coworkers studied the role of cations and anions in the growth inhibition and oxidative stress induced by four ILs in *S. obliquus*. The authors concluded that imidazolium-based ILs generates higher ROS level than pyridinium-based ILs. The order of the ILs regarding the anion effect on ROS is: [C_6_mpy][Cl] < [C_6_mpy][Br] < [C_6_mim][Cl] < [ C_6_mim][NO_3_], this also correspond to the order of their acute toxicity.

Two studies [86,92] have shown that when *S. obliquus* and *Chlorella pyrenoidosa* (*C. pyrenoidosa*) are exposed to low concentrations of IL, maximum toxicity is reached after 48 h. At the end of this exposure time, the plants can regain their normal levels of growth inhibition similar to those of the control.

Habibul and coworkers have performed studies on soil and water decontamination of ILs through plants. For ryegrass (*Lolium perenne* L.) [96], they observed that it could absorb more than 80% of [C_4_mim][Br]. This species revealed high effectiveness to phyto-extract the IL cation ([C_4_mim]) from water with a high root concentration factor, although the electron transfer from root to leaves seemed unfavored. The authors also performed a preliminary study of the [C_4_mim] metabolic pathway in ryegrass, concluding that the metabolic process of the cation with this species could occur on the tail of the cation alkyl chain. It was also observed a slight decrease in the chlorophyll content of ryegrass after a 15 day exposure. However, the increase of [C_4_mim] concentration caused a considerable decrease in chlorophyll content. As expected, significant growth inhibition of ryegrass was observed with the increase in the cation concentration. A similar study was performed in ryegrass using [C_2_mim][Br] and [C_8_mim][Br][97]. The uptake studies show that the uptake efficiencies of ILs were affected not only by their initial concentration but also by their alkyl chains. The higher the initial concentration of IL, the higher the inhibitory or toxic effect on the plant leading to a lower IL uptake rate. As previously observed, ILs accumulate more in roots than in other parts of the plant and [C_2_mim][Br] contents in ryegrass tissues were significantly higher than those of [C_8_mim][Br], again proving the influence of cation alkyl chain length. Later, the impact of the alkyl chain length in the uptake and accumulation of three ILs ([C_2_mim][Br], [C_4_mim][Br] and [C_8_mim][Br]) in rice seedlings was also studied by Habibul et al. [98]. Like in the previous study, the ILs were mostly accumulated in roots, and only a small amount was translocated and accumulated in stem and leaves. The chlorophyll content also decreased with the increase of ILs concentration, and this effect was higher for [C_8_mim][Br] than [C_4_mim][Br] and [C_2_mim][Br]. The growth inhibition was also higher in [C_8_mim][Br] than [C_2_mim][Br], confirming that the toxic effect of these ILs in plant growth increases with the cation alkyl chain length.

Xu et al. [99] study the toxic effect of three ILs ([C_6_mim][Br], [C_6_mim][NO_3_] and [C_6_mim][BF_4_]) in soil on *Vicia faba*. After 10 days of exposure to ILs, residue concentrations of the three ILs in soil decreased lightly. All the ILs were studied to inhibit the growth of *Vicia faba.* However, inhibition effects were higher for [C_6_mim][BF_4_] than [C_6_mim][Br] and [C_6_mim][NO_3_], where the inhibition effects were visible even at the lowest exposure dose (500 mg·kg^−1^). [C_6_mim][BF_4_] also presented considerably higher toxicity (EC_50_ of 1038.5 and 1126.8 mg·kg^−1^ for shoot length and dry weight, respectively) than [C_6_mim][Br] (EC_50_ of 2142.9 and 1802.5 mg·kg^−1^ for shoot length and dry weight, respectively) and [C_6_mim][NO_3_] (EC_50_ of 2124.5 and 1853.1 mg·kg^−1^ for shoot length and dry weight, respectively). Based on all the results presented in the work, the toxic order of these three ILs was: [C_6_mim][BF_4_] > [C_6_mim][Br] > [C_6_mim][NO_3_].

Since 2015 only a few studies about PILs toxicity and/or biodegradability have been published. Peric and coworkers [100] have predicted the toxicity of forty-five APILs and ten PILs using a quantitative structure–activity relationship (QSAR) model. In the case of the selected AILs, this method showed that the anion harms toxicity and the contribution of the cation was, as expected, positive, leading to increased toxicity. The size of the alkyl side-chain also influences the toxicity, and even the smallest side-chain presents a positive contribution. For the PILs, both anion and cation influence their toxicity, and in aquatic plants, growth inhibition, although the elongation of acid in the anionic moiety has more influence on toxicity. The authors also suggest that this model could help select and synthesize new and more sustainable ionic liquids. Oliveira et al. [71] studied the impact of four PILs on the germination of lettuce seeds (*Lactuca sativa*). The increase in the alkyl chain (and consequently of the hydrophobicity) lead to decreased IL concentration, which prevents the germination of lettuce seeds by 50% (LD_50_). The order of toxicity observed was: [OHC_2_C_1_NH_2_][CH_3_COO] (LD_50_ = 1.85 ± 0.01 > [OHC_2_C_1_NH_2_][C_2_H_5_COO] (LD_50_ = 1.18 ± 0.03) > [OHC_2_C_1_NH_2_][C_3_H_7_COO] (LD_50_ = 1.16 ± 0.01) > [OHC_2_C_1_NH_2_][C_4_H_9_COO] (LD_50_ = 0.45 ± 0.04). The authors observed with an optical microscope that all PILs caused changes in the cell structure. This impact was higher for the PILs with a longer alkyl chain ([OHC_2_C_1_NH_2_][C_3_H_7_COO] and [OHC_2_C_1_NH_2_][C_4_H_9_COO]), suggesting that the elongation of the alkyl chain has a strong effect on PILs toxicity as previously predicted by Peric et al. [100].

**Figure 8:**
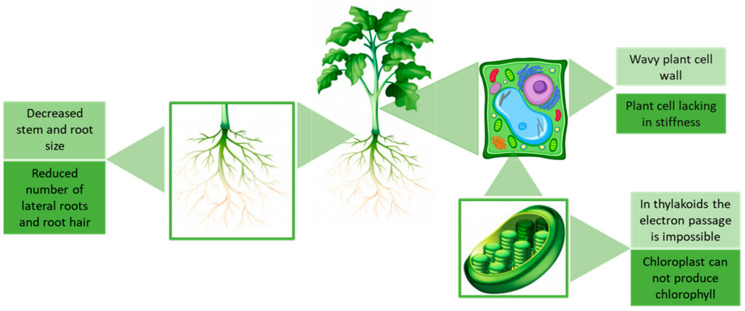
Schematic representation of the effect of ILs on plants.

In conclusion, the type and structure of the cation were the most responsible for their toxic effect on plants. The type of anion also contributed to the IL toxicity, although more studies are needed in vegetal systems. It is proven that ILs can be toxic to plants (from seedlings to bigger plants), affecting roots and stem. ILs can also enter the cells blocking the electron passage, the chlorophyll production and consequently the photosynthesis process and cell death (see Figure 8). Protic ionic liquids can be a “greener” alternative to the more studied ILs (imidazolium and pyridinium-based ILs), although more toxicological and biodegradability studies are needed.

## 4. Effect of Ionic Liquids on Animals

Ionic liquids can enter the environment through wastewater discharges and leaching of landfills, increasing the need to discover the effects of their contamination in aquatic and terrestrial ecosystems. The risk of the persistence of ILs in the environment and their effects on living organisms has led researchers to investigate the hazard they could present to compromised habitats before and indiscriminate use. The firsts toxicological trials focused mainly on the aquatic impact of ILs in organisms like fish, algae and crustaceous [101,102,103,104,105]. Over time the toxic effect of ILs in soil organisms (plants and animals) [106,107] was also studied as well as the enzymatic inhibition (acetylcholinesterase enzyme and cell lines). The most used ranking to classify the toxicologic behavior of compounds against aquatic organisms is the one defined by Passino and Smith [108] using the EC_50_ values expressed in μg·mL^−1^ (see Table 3).

*Danio rerio* (zebrafish) is a small freshwater fish recommended by the OECD as a bioindicator to evaluate de toxic effects of chemicals. Due to their availability, low cost, easy maintenance and rapid development of transparent embryos, this fish has been widely used to determine ILs toxicity on aquatic organisms. The first study of the toxicity of 1-octyl-3-methylimidazolium chloride ([C_8_mim][Cl]) and 1-octyl-3-methylimidazolium tetrafluoroborate ([C_8_mim][BF_4_]) on zebrafish liver cells was presented by Liu et al. [109]. Exposure to toxic chemicals can cause an imbalance in the levels of reactive oxygen species (ROS), including superoxide radicals (O_2_•^−^), hydrogen peroxide (H_2_O_2_), hydroxyl radicals (OH•^−^) and peroxide radicals (RCOO). The preservation of certain concentrations of ROS is essential for an organism, although the excess of ROS tends to create stress in the cell and modify cellular redox processes causing damage in proteins and DNA, changes in the shape of the cell and even among many other symptoms, cell death. In the study, the increase in these ILs concentrations increased ROS levels in the liver cells and stayed high with time, causing oxidative damage even at the lowest concentration (5 mg·L^−1^) on the 7th day. At high concentrations, [C_8_mim][Cl] and [C_8_mim][BF_4_] can cause the zebrafish death. It was also observed that the anions might have a small impact on the toxicity.

Zebrafish were also used in the study [110] of the acute toxicity of 1-alkyl-3-methylimidazolium nitrate ([C_n_mim][NO_3_] with *n* = 2, 4, 6, 8, 10, 12) and 1-hexyl-3-methylimidazolium ILs ([C_6_mim][R] where R = [Cl], [Br], [BF_4_], [PF_6_]). The 50% lethal concentration (LC_50_) was utilized to determine the acute toxicity of the ILs. [C_2_mim][NO_3_] was the least toxic IL with LC_50_ values surpassing 3000 mg L^−1^ and can be considered relatively harmless, according to Passino and Smith [108]. [C_4_mim][NO_3_], [C_6_mim][NO_3_], [C_6_mim][Br], [C_6_mim][Cl], [C_6_mim][BF_4_] and [C_6_mim][PF_6_] had LC_50_ values between 120 and 867 mg·L^−1^ and are classified as practically harmless. [C_8_mim][NO_3_] was considered slightly toxic (LC_50_ varied between 23.1 and 35.6 mg·L^−1^), while [C_10_mim][NO_3_] and [C_12_mim][NO_3_] had the lowest LC_50_ values (inferior to 10) and were classified as moderately toxic. This study also observed that the anions had a small impact on ILs toxicity since ILs with different anions had similar LC_50_ values. As usual, increased the cation alkyl side-chain length originated increased ILs toxicity. A further study of the 1-methyl-3-hexylimidazolium bromide ([C_6_mim][Br]) toxicity on zebrafish [111] demonstrated that this IL had toxic effects on zebrafish, inducing an increase in the ROS levels that causes DNA damage. The effect of the gender was also investigated, and no significant differences were observed, and a 1:1 male-female zebrafish ratio can be used in this type of study. Another study [112] from the same laboratory about acute toxicity of [C_4_mim][BF_4_] and 1-butyl-3-methylimidazolium chloride ([C_4_mim][Cl]) in liver cells of zebrafish revealed that these ILs could be considered practically harmless, with LC_50_ values (after 96 h of exposure) of 604.6 ± 56.2 mg·L^−1^ and 632.8 ± 67.4 mg·L^−1^, respectively. The small discrepancy of these values indicates a minor effect of the anion. As observed in previous studies, the ROS levels increased with the increase of the IL concentration apart from a slight decrease at 40 mg·L^−1^. This may suggest that ROS levels could be maintained balanced at small doses of IL. Antioxidant enzymes eliminate the ROS excess like superoxide dismutase (SOD) and catalase (CAT), where SOD catalyzes the conversion of superoxide anions to hydrogen peroxide eliminated by CAT. The increase of ROS levels on day 7 induced the SOD activity and inhibited it on day 14, suggesting that the oxidative stress was not repaired quickly by SOD. At high concentrations (160 mg·L^−1^), the CAT was inhibited during the entire exposure period that could be justified by the excess of hydrogen peroxide induced by SOD. The DNA damage increased with the increase of ILs concentration, showing that the DNA strand breaks in the zebrafish liver as a result of the [C_4_mim][BF_4_] and ([C_4_mim][Cl] genotoxicity. The effect of anion and cation alkyl side-chain of 1-alkyl-3-methylimidazolium-based ILs on zebrafish was also studied [113]. In this work, 1-ethyl-3-methylimidazolium ([C_2_mim]) with three different anions ([Cl], [Br] and [BF_4_]) were used, as well as 1-alkyl-3-methylimidazolium bromine ([C_n_mim][Br]) with n = 2, 4, 8, 10, 12. It was observed that the cumulative mortality was dose-dependent and time-dependent, i.e., the higher the IL concentration dose and longer the exposure time were, the higher the cumulative mortalities. Although some exceptions were observed in 3000 mg·L^−1^ of [C_2_mim][Cl] at 72 h and 3000 mg·L^−1^ of [C_2_mim][BF_4_] at 48 h. The acute toxicity of ILs increased with the increase of the cation alkyl side-chain length following the trend [C_2_mim] (relatively harmless) < [C_4_mim] (practically harmless) < [C_8_mim] (slightly toxic) < [C_10_mim] (moderate toxic) < [C_12_mim] (moderate toxic). For the 96 h LC_50_ values of the different anions, [C_2_mim][Br] (96 h LC_50_ = 2970 mg·L^−1^) was the less toxic, followed by [C_2_mim][Cl] (96 h LC_50_ = 2620 mg·L^−1^) and the [C_2_mim][BF_4_] (96 h LC_50_ = 2170 mg·L^−1^), still all of them are classified as relatively harmless. A small discrepancy is observed when comparing this result to the one obtained by Liu et al. [114] for *Vicia faba* with [C_8_mim] ([Cl], [Br] and [BF_4_]), with the toxicity scale being: [BF_4_] > [Br] > [Cl]. This laboratory also has recently presented [115] the effect of 1-aminoethyl-3-methylimidazolium tetrafluoroborate ([NH_2_C_2_mim][BF_4_]), 1-methoxyethyl-3-methylimidazolium tetrafluoroborate ([C_1_OC_2_mim][BF_4_]) and 1-hydroxyethyl-3-methylimidazolium tetrafluoroborate ([HOC_2_mim][BF_4_]) on zebrafish. The acute toxicity study indicated that [HOC_2_mim][BF_4_] with 96 h LC_50_ = 3086.7 mg·L^−1^ was the less toxic, followed by [C_1_OC_2_mim][BF_4_] (96 h LC_50_ = 2492.5 mg·L^−1^) and [NH_2_C_2_mim][BF_4_] with 96 h LC_50_ = 143.8 mg·L^−1^. The less toxic ILs can be classified as relatively harmless, and the [NH_2_C_2_mim][BF_4_] can be classified as practically harmless. To observe the effects of theses ILs on antioxidant enzyme activities (SOD, CAT and glutathione S-transferase (GST)), zebrafish were exposed for 7, 14, 21 and 28 days, to [NH_2_C_2_mim][BF_4_] (0, 5, 10, 20 and 40 mg·L^−1^), [C_1_OC_2_mim][BF_4_] (0, 1, 10, 50 and 100 mg·L^−1^) and [HOC_2_mim][BF_4_] (0, 1, 10, 50 and 100 mg·L^−1^). All the ILs induced increased ROS and malondialdehyde (MDA) levels causing the inhibition of SOD activity (especially when zebrafish was exposed to [NH_2_C_2_mim][BF_4_] and [HOC_2_mim][BF_4_]) and DNA damage was induced. Based on the integrated biomarker response the toxicity order was: [C_1_OC_2_mim][BF_4_] > [HOC_2_mim][BF_4_] > [NH_2_C_2_mim][BF_4_].

Perales et al. [116] compared the toxicity of 1-butyl-3-methylimidazolium hexafluorophosphate ([C_4_mim][PF_6_]) with 3-bis(2,2,2-trifluoroethoxy)propan-2-ol (BTFIP) against three aquatic organisms: the bacterium *V. fischeri*, the crustaceous *Daphnia magna* (*D. magna*) and the fish *Danio rerio*. Regarding the effect on *V. fischeri*, the EC_50_ values for both solvents are very similar, [C_4_mim][PF_6_] (EC_50_ = 1473 mg·L^−1^) and BTFIP (EC_50_ = 1597 mg·L^−1^), although none of them could be considered as toxic. In the case of *D. magna*, both of the solvents affected its mobility. Still, the IL (EC_50_ = 31 mg·L^−1^) was more toxic than the BTFIP (EC_50_ = 477 mg·L^−1^), and consequently, the [C_4_mim][PF_6_] can be classified as moderately toxic, while BTFIP can be considered as practically harmless. The *Danio rerio* studies revealed that both solvents have similar lethal concentrations, but contrary to what was observed in *V. fischeri*, the [C_4_mim][PF_6_] was less toxic than the BTFIP with LC_50_ values of 550 and 353 mg·L^−1^, respectively, and both of them can be considered as practically harmless. Still, for concentrations of BTFIP higher than 2000 mg·L^−1^, dead fish were observed after the first hour, and strange behavior occurred when the fishes were exposed to BTFIP concentrations of 500 and 750 mg·L^−1^.

Ruokonen et al. [117] studied the effect of eleven imidazolium, phosphonium and amidinium-based ILs on zebrafish and Chinese hamster ovary cells (CHO). For zebrafish embryos, [P_4,4,4,1_][C_9_H_19_COO] and [P_8,4,4,4_][CH_3_COO] were not at all toxic for a concentration of 1 mg·L^−1^, but killed all embryos at 100 mg·L^−1^ after 24 h and 120 h, respectively, taking into account the onset of death for these two ILs, [P_4,4,4,1_][C_9_H_19_COO] even infiltrates the embryo membrane. Even more toxic, ([P_14,4,4,4_][CH_3_COO]) is fatal to all specimens in the space of 10 min at 100 mg·L^−1^, and at 1 mg·L^−1^, around 0 to 5 fish of the initial 30 are left alive after 5 days (EC_50_ < 1 mg·L^−1^), indicating high acute toxicity. It was also observed that the most lethal ILs caused malformations. When CHO cells were also used to determine EC_50_ values, the results revealed the two most toxic ILs were [P_14,4,4,4_][CH_3_COO] (EC_50_ = 2.5 ± 0.85 mg·L^−1^) and [P_8,4,4,4_][CH_3_COO] (EC_50_ = 54 ± 2 mg·L^−1^) and can be classified as toxic and moderate toxic, respectively. The decrease of the alkyl chain length in the cation revealed decreased IL toxicity, while the increased alkyl chain length of the anion seemed to have a much lower influence on the toxicity (Table 4).

Baharuddin and coworkers [118] studied the ecotoxicity of six amino acid ILs against zebrafish. The selected cations were tetrabutylphosphonium ([P_4,4,4,4_]), tetrabutylammonium ([N_4,4,4,4_]) and cholinium ([Cho]) and the anions chosen were the phenylalanine ([Phe]) and taurine ([Tau]). The acute toxicity studies indicated that all the ILs synthesized had 96 h LC_50_ values higher than 100 mg·L^−1^ and would be classified as practically harmless.

Other examples of ILs versatility are the active pharmaceutical ingredients-ionic liquids (API-ILs). The study of the adverse impact of API-ILs on aquatic ecosystems was presented by Tang and coworkers [119], where several API-ILs based on triflumizole (a broad-spectrum systemic fungicide) were synthesized. All the ILs synthesized were insoluble in water but highly soluble in methanol. The 96 h LC_50_ values were extremely low (varied between 2.20 and 4.40 mg·L^−1^) and could be classified as moderately toxic. The toxicity order of these API-ILs with the different anions was: lactic acid > nicotinic acid > oleic acid > pelargonic acid > acetic acid > salicylic acid. Solvated ionic liquids are a class of ILs where their cationic portion consists of two components themselves. The toxicity study [120] of equimolar solutions of lithium bistrifluoromethylsulfonimide in triglyme (G3TFSA) or tetraglyme (G4TFSA) showed that when zebrafish embryos were exposed (24–50 h) to these ILs, the levels of cell deaths were very low and moderate apoptosis only occurred at the highest concentration (100 µM). The G4TFSA was more toxic than G3TFSA, although their toxicity was very low. These ILs could be a safe alternative to common solvents like dimethyl sulfoxide (DMSO). Due to these promising results, the authors suggest that G3TFSA and G4TFSA could be used as solvents for pharmaceutical compounds normally used in research laboratories and even replace DMSO for in vitro and in vivo experimental research.

Younes et al. [121] studied the effect of choline chloride ([Cho][Cl]), 1-methyl-1-propylpyrrolidinium trifluoromethanesulfonate ([C_3_mpyr][CF_3_SO_3_]) and tetramethylammonium acetate ([N_1,1,1,1_][CH_3_COO]) on the embryonic development of zebrafish, for their possible application as hydrate inhibitors in the oil and gas industries. No mortality on zebrafish embryos was observed even at the highest concentration (200 mg·L^−1^). The study on motility showed that 200 mg·L^−1^ of [Cho][Cl] induce a relevant increase in tail coiling activity compared to the negative control, while [C_3_mpyr][CF_3_SO_3_] and [N_1,1,1,1_][CH_3_COO] did not elicit any significant effect in tail coiling activity. This suggests that [Cho][Cl] could harm the nervous system of zebrafish embryos. The embryos exposure to 200 mg·L^−1^ of [Cho][Cl] and [N_1,1,1,1_][CH_3_COO] caused decreased hatching rates and delayed the hatching times. On [Cho][Cl] it was also observed a dose-dependent effect. At low concentrations (50 and 100 mg·L^−1^) of [Cho][Cl] and [N_1,1,1,1_][CH_3_COO] most embryos were still viable at 96 h of exposure. Based on these results on hatchability, the [Cho][Cl] and [N_1,1,1,1_][CH_3_COO] could harm embryo survival rates at a later stage of their life, mainly at high concentrations. [C_3_mpyr][CF_3_SO_3_] did not induce any toxic effect on zebrafish embryos.

Perez et al. [122] used desorption electrospray ionization–mass spectrometry imaging (DESI-MSI) to evaluate the accumulation of an AMMOENG (ILs composed by tetrasubstituted-ammonium cations with varying saturated alkyl or oligo(ethyleneglycol) chains and small anions like [Cl] or [CH_3_OSO_3_]) on zebrafish. The zebrafish were exposed to three concentrations (1.25, 2.5 and 5.0 mg L^−1^) of AMMOENG 130 (dimethyldioctadecylammonium chloride ([N_1,1,18,18_][Cl])) for 96 h. After 24 h, the zebrafish exposed to 2.5 and 5 mg·L^−1^ started to display signs of acute toxicity like inflammation of the gills and buccal cavity, epidermal discoloration, abdominal distension and inflammation. Through the DESI-MSI analysis of the zebrafish gills, it was possible to observe the appearance of a dealkylated IL metabolite (trimethylstearylammonium ion) in the lowest concentration of exposure. Accumulated in the nervous and respiratory systems, the AMMOENG 130 and the metabolite could penetrate the blood–brain barrier (significant accumulation in the brain was observed). This method appears to be a promising technique to acquire information on the characterization, distribution and metabolism of an IL in zebrafish all at once.

Thamke and Kodam [123] used *Poecilia reticulata* (guppy fish) to determine the toxicity of 1-butyl-3-methylimidazolium bromide ([C_4_mim][Br]). When exposed to this IL, the SOD activity increased with the concentration up to 50 mg·L^−1^ and suddenly decreased at 100 mg·L^−1^, probably due to a decrease of ROS species. The same behavior was observed with the CAT activity. The genotoxicity study revealed that the increase in IL concentration from 50 to 100 mg·L^−1^ exacerbated DNA damage in the liver cells. Xa and coworkers [124] found that long-term exposure of *Hypophthalmichthys molitrix* (silver carp) to 1-methyl-3-octylimidazolium bromide ([C_8_mim][Br]) caused organ damage, especially in fish liver. The acid phosphatase and alanine aminotransferase activities and the serum lactate dehydrogenase levels increased with the increase of IL concentration and time of exposure, causing oxidative stress in the carp organs. The chronic exposure of this IL in the carp liver caused an inflammatory reaction, inducing hepatic apoptosis via the mitochondria pathway.

The marine mussel *Mytilus galloprovincialis* was used to investigate [125] the toxicity of 1-butyl-3-methylimidazolium tetrafluoroborate ([C_4_mim][BF_4_]) and 1-methyl-3-octylimidazolium tetrafluoroborate ([C_8_mim][BF_4_]). It was also evaluated if acetone could mediate the ILs toxic profile. In the 96 h mortality test, it was observed that adding acetone to [C_4_mim][BF_4_] increased the IL mortality at concentrations higher than 10 mg·L^−1^. The [C_8_mim][BF_4_] was revealed to be more toxic to mussel even at lower concentrations, but the presence of acetone resulted in a slight decrease of its toxicity up to a concentration of 100 mg·L^−1^. The neutral red retention assay (NRRT) values in hemocytes were practically the same with or without acetone. In the [C_8_mim][BF_4_] treated mussels, the NRRT values were lower than the ones obtained for [C_4_mim][BF_4_]. The SOD activity was practically the same on mussels treated [C_4_mim][BF_4_] with or without acetone with a small increase of ROS content, while [C_8_mim][BF_4_] a significant increase of ROS content were observed, at concentrations of 0.1 to 0.5 mg·L^−1^. Genotoxic effects of [C_4_mim][BF_4_] were negligible with or without acetone at lower concentrations, while high levels of DNA damages were observed in the [C_8_mim][BF_4_] at lower concentrations (without acetone—0.01 and 0.1 mg·L^−1^, with acetone—0.1 and 0.5 mg·L^−1^). A further study [126] showed that [C_8_mim][BF_4_] (concentrations from 0.7 to 1.75 µM) could induce cytotoxicity in *Mytilus galloprovincialis* hemocytes, causing almost 50% reduction of cell viability for concentrations higher than 3.5 µM, increased the ROS species and lipid peroxidation byproducts and caused increasing levels of DNA damage.

The toxicity effect of ILs on small aquatic crustaceans (like *Daphnia magna*, *Artemia salina*¸ and others) has also been studied. Siciliano et al. [127] study the toxic effect of imidazolium-based ILs on *Daphnia magna* (*D. magna*). The results showed a progressive decrease in the survival of daphnids when exposed to 1-ethyl-3-methylimidazolium chloride ([C_2_mim][Cl]) in the first 6 days, and only 60% of daphnids survived after 21 days. The exposure to 1-butyl-3-methylimidazolium chloride ([C_4_mim][Cl]) shows a 50% survival rate. The analysis of ILs byproducts revealed that successive generations presented toxic effects on daphnids up to the third generation. Both ILs and their byproducts increased the biochemical stress in daphnids due to the degradation of their chemical pathways. It was also observed that these ILs could be more toxic than imidazole. In the work of Zhang et al. [128], the toxicity effect of six 1-alkyl-3-mthylimidazolium nitrate ILs ([C_n_mim][NO_3_] with n = 2, 4, 6, 8, 10, 12) on *D. magna* was evaluated during 24 and 48 h of exposure. It was observed that the increase of concentration caused the increase of cumulative immobilization after 24 h and 48 h, except for the 45 mg·L^−1^ of [C_2_mim][NO_3_], 2.5 mg·L^−1^ of [C_4_mim][NO_3_] and 0.25 mg·L^−1^ of [C_8_mim][NO_3_] at 24 h of exposure. In the 48 h cumulative immobilization, all the ILs reached 100% for the highest concentration. The 48 h LC_50_ values demonstrated that [C_2_mim][NO_3_] (LC_50_ = 48.0 mg·L^−1^) is the less toxic IL and can be classified as slightly toxic and the toxicity increased with the increase of the cation alkyl side-chain length where [C_12_mim][NO_3_] (LC_50_ = 0.012 mg·L^−1^) was the most toxic IL and can be classified as highly toxic as well as [C_6_mim][NO_3_] (LC_50_ = 0.36 mg·L^−1^), [C_8_mim][NO_3_] (LC_50_ = 0.27 mg·L^−1^) and [C_10_mim][NO_3_] (LC_50_ = 0.027 mg·L^−1^). The [C_4_mim][NO_3_] had an LC_50_ of 0.36 mg·L^−1^ and can be classified as moderately toxic. Despite these ILs being relatively stable in aquatic environments for a short period, all of them presented potential risk if they were accidentally disposed of in the aquatic environment. Combining the OECD *D. magna* standard test and fish immunomarkers has been suggested for the complete examination of ILs impact in different organisms and trophic levels [129]. The toxicity of 8 phosphonium and imidazolium-based ILs on *D. magna* was evaluated by the new method. The immobilization test revealed that three tri(hexyl)tetradecylphosphonium ILs were more toxic than the other ILs, with EC_50_ values inferior to 0.5 mg·L^−1^. The less toxic IL was the 1-ethyl-3-methylimidazolium ethylsulfate ([C_2_mim][C_2_H_5_OSO_3_]) with EC_50_ = 300.8 mg·L^−1^ and the most toxic IL was the tri(hexyl)tetradecylphosphonium chloride ([P_6,6,6,14_][C_2_H_5_OSO_3_]) with EC_50_ = 0.053 mg·L^−1^. The tri(isobutyl)methylphosphonium tosylate ([P_i4,i4,i4,1_][TOS]) was the less toxic of the phosphonium-based ILs with EC_50_ = 130.7 mg·L^−1^. Regarding leukocyte distribution, divergent effects among the two sets of ILs were observed. The imidazolium-based ILs showed a decreasing trend of the granulocyte-macrophage percentage, while phosphonium-based ILs tended to reduce the percentage of lymphocytes and appear to cause larger changes to leukocyte distribution. Both classes of ILs cause an expressive increase in total cellular mortality, apoptosis and necrosis percentages. Imidazolium-based ILs caused a significant decrease in lysosomal membrane integrity and phagocytosis activity at higher concentrations. The authors argued that since ILs are in continuous development, the combination between a standardized test and a mechanism of the action-based test appears to be the more coherent way to evaluate the ILs toxicity.

*Artemia salina* has also been used in the toxicological study of ILs, Bioucas et al. [130] observed that [C_2_mim][CH_3_SO_3_] (LC_50_ = 4.834 mg·L^−1^) was more toxic than ethylene glycol, and this IL could be classified as highly toxic, although authors suggest that more studies are needed for a better analysis of [C_2_mim][CH_3_SO_3_] toxicity. The brine shrimp lethality test (BSLT) was used to determine the toxicity of several ILs on *Artemia salina* cysts [131]. It was observed that an increase of IL concentration provoked decreased *Artemia salina* hatchability. The choline chloride ([Cho][Cl]) and choline acetate ([Cho][CH_3_COO]) were the less toxic ILs. Besides the cation, choline had a positive effect on the toxicity. However, when the anion was changed to dihydrogen phosphate ([H_2_PO_4_]), the hatching process was inhibited. Similar toxicities were observed for 1-butyl-3-methylimidazolium nitrate [C_4_mim][NO_3_], 1-butyl-3-methylimidazolium acetate [C_4_mim][CH_3_COO] and methylammonium nitrate [CH_3_NH_3_][NO_3_]. The toxicity of these ILs followed the order [Cho][H_2_PO_4_] > [C_4_mim][NO_3_] > [C_4_mim][CH_3_COO] > [Cho][CH_3_COO] ≥ [Cho][Cl]. The hatchability is affected by the pH of the solution, and it was observed that low pH causes low hatchability. When the pH of the solution with [Cho][H_2_PO_4_] is adjusted to 7, the EC_50_ values became similar to those of [Cho][Cl]. The toxic effect of a series of sixteen PILs was evaluated against *Artemia salina* nauplii [132]. The medium lethal concentration (LC_50_) was determined after 24 h of incubation. It was observed that all the PILs synthesized were considered nontoxic, except hydroxyethanaminium butyrate and N-methyl-2-hydroxy-ethyl-ammonium butyrate that was classified as weakly toxic and hydroxyethyl-ammonium hexanoate that was considered moderately toxic.

The deterioration of an aquatic ecosystem can be monitored by observing freshwater planarians since they are highly sensitive to low concentrations of environmental contaminants and can be easily collected in large numbers and kept in a laboratory. *Dugesia japonica* (*D. japonica*) is a common species of the freshwater planarian. Zhang and coworkers studied the toxic effect of 1-octyl-3-methylimidazolium bromide ([C_8_mim][Br]) on this planarian [133,134,135], using the randomly amplified polymorphic DNA (RAPD) assay, DNA damage analysis can contribute to understanding the toxicity mechanisms of ILs on planarians. Based on the results, the authors found that this IL showed genotoxic effects on *D. japonica* and that the antioxidant enzyme activities were also modified, causing oxidative damage. The studies [136] of the alkyl side-chain effect using 1-alkyl-3-mthylimidazolium bromide ([C_n_mim][Br] with n = 4, 6, 8, 10) against *D*. *japonica* revealed a strong effect on antioxidant enzymes and glutathione (GSH) levels, triggering the antioxidant system to protect these planarians from an excess of ROS species.

The role of acetone in the induced toxicity of two 1-alkyl-3-methylimidazolium tetrafluoroborate ([C_n_mim][BF_4_] with n = 4, 8) was studied [137] against the algae *Scenedesmus rubescens*, crustaceans *Thamnocephalus platyurus* and *Artemia franciscana*, rotifers *Brachionus calyciflorus* and *Brachionus plicatilis* and bivalve *Mytilus galloprovincialis*. The algae growth rate was notably lower in [C_8_mim][BF_4_] than the ones obtained in control, while [C_4_mim][BF_4_] only had a lower growth rate at higher concentrations (100 and 200 mg·L^−1^) for 24 h. The acetone-free binary mixture of these ILs had a rapid growth inhibition over time, even higher than [C_8_mim][BF_4_]. According to EC1272/2008 Regulation [138] and the IC_50_ values, the [C_8_mim][BF_4_] and the ILs binary mixture can be classified as hazardous to the aquatic environment (acute category IC_50_ ≤ 0.1 mg·L^−1^). Freshwater species (*Thamnocephalus platyurus* and *Brachionus calyciflorus*) were more sensitive than the estuarine/marine species (*Artemia fransciscana*, *Brachionus plicatilis* and *Mytilus galloprovincialis*) in all cases. Regarding *Thamnocephalus platyurus*, [C_4_mim][BF_4_], acetone–[C_8_mim][BF_4_] and acetone–ILs binary mixture can be classified as moderately toxic, while [C_8_mim][BF_4_] and the ILs binary mixture were classified as highly toxic. Regarding *Brachionus calyciflorus*, [C_4_mim][BF_4_] was slightly toxic, acetone–[C_8_mim][BF_4_] was moderately toxic, [C_8_mim][BF_4_] and the ILs binary mixtures with and without acetone were classified as highly and very highly (extremely toxic), respectively for 24 h and 48 h of exposure. In the case of *Brachionus plicatilis*, [C_4_mim][BF_4_] (with and without acetone) were practically nontoxic, acetone–[C_8_mim][BF_4_] and the acetone–ILs binary mixture were classified as slightly toxic, while [C_8_mim][BF_4_], the binary mixture and acetone–[C_4_mim][BF_4_] were classified as moderately toxic. In *Artemia franciscana*, toxic effects were not observed for the [C_4_mim][BF_4_] and the binary mixtures with or without acetone, and only [C_8_mim][BF_4_] could be classified as slightly toxic. In the case of mussels, [C_4_mim][BF_4_] and acetone–[C_4_mim][BF_4_] were classified as practically nontoxic and slightly toxic, respectively. Comparatively, [C_8_mim][BF_4_] and acetone–[C_8_mim][BF_4_] could be classified as high and moderately toxic, respectively. The ILs binary mixture, with and without acetone, was classified as moderately toxic.

The quantitative structure–activity relationship (QSAR) models can also be applied to predict the toxicities of ILs toward aquatic organisms. Liu et al. [139] developed four norm-index QSAR models for the acute toxicity of ILs toward zebrafish. The four models retrieved good results with R^2^ of 0.8549, 0.9162, 0.8335 and 0.8119 for pLC_50_-48 h, pLC_50_-96 h, pLC_50_-120 h and pLC_50_-132 h, respectively. Cho et al. [140] developed a simple toxicity prediction method correlating the experimental data from an insect cell line (*Spodoptera frugiperda* 9), an earthworm (*Eisenia fetida*), a nematode (*Caenorhabditis elegans*) and a fish (zebrafish) with the calculated values and determined linear coefficients. The R^2^ values for the earthworm, nematode and fish were 0.88, 0.96, and 0.94–0.95, respectively. For the insect cell line, the calculated parameters were correlated with log 1/LC_50_ values after 24 and 48 h of incubation, and the R^2^ were 0.67 and 0.88, respectively. The same authors use linear free energy relationship (LFER) descriptors to explain the toxicity of *Daphnia magna* and developed a new reasonable model with an R^2^ = 0.867 for the overall set (training set had an R^2^ = 0.880 and the test set an R^2^ = 0.848) [141]. A QSAR model was developed to predict the toxicities of ILs against *Caenorhabditis elegans* (*C. elegans*). This model used the experimental data of thirty imidazolium-based ILs, and the LC_50_ values were log-transformed (lg LC_50_) and presented an R^2^ = 0.938 [142]. All these models had the potential to help in the design and selection of new ionic liquids with low toxicity to the environment and human health.

The ionic liquids’ impact on soil contamination should also be considered when studying the ILs toxicity. Earthworms are the most studied organisms due to their essential role in nutrient mineralization, decomposition and soil structure improvement. *Eisenia fetida* (*E. fetida*) is one of the most studied earthworms in evaluating the ILs effect on soil ecosystems and is one of the recommended specimens by the Organization for Economic Cooperation and Development (OECD) in their guidelines for the testing of chemicals. Shao et al. [143] analyzed the toxic effect of [C_8_mim][BF_4_] and 1-octyl-3-methylimidazolium bromide ([C_8_mim][Br]) on *E. fetida* where a concentration of 5 mg kg^−1^ did not increase the ROS content, while higher concentrations (>10 mg kg^−1^) revealed a significant increase of ROS content. SOD activity was stimulated after ILs exposure at lower concentrations (5 and 10 mg·kg^−1^) for the different exposure periods, except the 5 mg·kg^−1^ concentration of [C_8_mim][BF_4_] at 28 days of exposure. It was also observed a reduction in the SOD activity to or below the control levels for the 20 and 40 mg·kg^−1^ ILs concentrations at 21 and 28 days of exposure for [C_8_mim][BF_4_] and at 7 and 14 days of exposure for [C_8_mim][Br]. The CAT activity in [C_8_mim][BF_4_] did not significantly change compared with the control for 7 and 14 days of exposure. In [C_8_mim][Br], changes in the CAT activity are visible and increase with increased concentration and exposure time. The peroxidase (POD) activity in [C_8_mim][Br] increases with concentration at 7 days of exposure, but with the increase of exposure time, this activity tends to decrease. This behavior was similar in [C_8_mim][BF_4_], except for 5 mg·kg^−1^ concentration at 7 days. The GST (detoxifying enzyme) activity increases with the increase of concentration and time of exposure for both ILs. No significant changes were observed in MDA content for [C_8_mim][Br], and in the case of [C_8_mim][BF_4_], the changes are only visible at 21 and 28 days of exposure. The olive tail moment (OTM) increases with time and concentration, suggesting that DNA damage depends on both time and dose exposure to ILs. The evaluation [144] of the toxicity of 1-octyl-3-methylimidazolium chloride ([C_8_mim][Cl]) on *E. fetida* showed a dose dependence of the ROS levels since they significantly increase with the increasing of the concentration until 21 days of exposure. At 28 days of exposure, the ROS levels at different concentrations are very similar. SOD and POD activities showed to be more sensitive at 7 through 14 exposure days. No significant differences in MDA were observed with IL concentrations and time. At 28 days of exposure, the MDA content slightly increased. The OTM values increased with increasing IL concentration and exposure time, indicating that the degree of DNA damage also increased, especially for concentrations higher than 5 mg·kg^−1^. The ecotoxicity of [C_8_mim][BF_4_] to earthworms was also studied in two types of soil: fluvo-aquic soil and artificial soil [145]. The acute toxicity of this IL was higher in fluvo-aquic soil (7d LC_50_ = 744 mg kg ^−1^ and 14d LC_50_ = 489 mg·kg ^−1^) than in the artificial soil (7d LC_50_ = 870 mg·kg ^−1^ and 14d LC_50_ = 679 mg·kg ^−1^). ROS levels were higher in artificial soil after 7 days than in fluvo-aquic soil, but at 28 days exposure, the ROS levels were higher in fluvo-aquic soil than in artificial soil and the response of SOD activity was better in fluvo-aquic soil. CAT activity significantly affected artificial soil, although an inhibitory effect in fluvo-aquic soil was observed. In artificial soil, high guaiacol POD activity was observed at 7 day exposure, but 7 days later, the activities were similar to the control, and after 28 days, a slight inhibition was observed. In fluvo-aquic soil, the POD activity was visibly higher than control at 14 days of exposure, although, at 21 days, these activities were significantly inhibited. GST activity was inhibited at the second half of the experiment for artificial soil, but this inhibitory effect occurred earlier in the fluvo-aquic soil. Regarding the OTM values, the increase was similar for both soils. However, the DNA damage was more noticeable in the fluvo-aquic soil. The toxic effects of the cation side-chain length of 1-alkyl-3-methyl imidazolium nitrate ([C_n_mim][NO_3_] with n = 4, 6, 8, 10, and 12) ILs to earthworms were evaluated by Shao et al. [146,147]. In these studies, the ROS content increased with the increase of the concentration and time, while the toxicity of these ILs follows the order [C_10_mim][NO_3_] < [C_12_mim][NO_3_] < [C_4_mim][NO_3_] < [C_6_mim][NO_3_]< [C_8_mim][NO_3_] (cutoff effect at n=8). The most notorious changes occurred after the exposure to [C_8_mim][NO_3_]. The greatest oxidative damage also happened after earthworms had been exposed to [C_8_mim][NO_3_]. The lipid oxidative damage product MDA concentrations increased with the increasing of the concentration and exposure time, showing to be dose and time-dependent. The OTM values also increased with concentration, and [C_8_mim][NO_3_] shows the highest values, while [C_10_mim][NO_3_] and [C_12_mim][NO_3_] had the lowest and very similar values. The same authors performed a similar study, but instead of using the anion nitrate, the bromide anion was selected. In studying the 1-alkyl-3-methyl imidazolium bromide ([C_n_mim][Br] with n = 2, 4, 6, 10 and 12) ILs toxicity to *E. fetida* [148], the analysis of the LC_50_ revealed an increase with increasing alkyl side-chain length up to [C_10_mim][Br] and then a small decrease. The ROS content also increased with increasing alkyl side-chain length, and consequently, the [C_2_mim][Br] was the least toxic, while the [C_12_mim][Br] was the most effective ROS inducer. The antioxidant enzymes and the detoxification enzyme GST presented similar results as all were trying to eliminate the damage caused by excess ROS. Oxidative damages had occurred in all ILs.

*Caenorhabditis elegans* (*C. elegans*) is a nematode that lives in temperate soil environments and is widely studied in several fields from genetics to toxicology, neurobiology or biology. The study [149] of the 1-ethyl-3-methylimidazolium bromide ([C_2_mim][Br]) toxicity on *C. elegans* eggs revealed that this IL provokes a significant stimulation on the initial reproduction, but a notorious inhibition on total reproduction overall, the lifespan was also affected by this IL. The initial reproduction of nematode adults was inhibited by [C_2_mim][Br], like the total reproduction and the lifespan. These results show that the younger nematodes are much more sensitive to the stimulatory effects of the IL. The inhibition of total reproduction and stimulation of the lifespan in the egg-stage data suggested a tradeoff relation, being considered as a potential survival strategy to environmental stresses. Regarding the eggs, the presence of the IL provoked decreased total ROS and H_2_O_2_ and increased O_2_•^−^ and OH•^−^ levels. SOD and CAT activities surged at higher concentrations but inhibited at the lowest concentration. On the adult nematodes, the increase in OH•^−^ and H_2_O_2_ (at higher concentrations) was significantly lower than for the eggs, and O_2_•^−^ was inhibited, while the most significant change in antioxidant behavior in the adults was the spike in oxidized glutathione. [C_2_mim][Br] induced life stage-dependent changes on *C. elegans*. This nematode was also exposed to different concentrations of 1-tetradecyl-3-methylimidazolium bromide ([C_14_mim][Br]) [150]. After exposure to this IL, no significant changes were observed in the body length for concentrations up to 0.5 mg·L^−1^. The prolonged exposure revealed a dose-dependent reduction. Similar results were observed concerning head thrashes and body bends. However, for IL concentrations higher than 2 mg·L^−1,^ it also revealed a dose-dependent reduction. Retardation of the development of the nematode larvae was observed mainly after 48 h of exposure to 5 and 10 mg·L^−1^ of [C_14_mim][Br]. No significant changes were observed in ROS levels and lipofuscin content after long exposure of the nematodes to [C_14_mim][Br] even at higher concentrations. The prolonged exposure to 5 and 10 mg·L^−1^ of [C_14_mim][Br] did not cause significant changes in SOD activity, suggesting that oxidative stress possibly is not the cause for the negative effects observed at low concentrations. A significant decrease in body length caused by RNAi-mediated inhibition of *pgp*-13 after long exposure to IL was observed. At higher concentrations, the effects were even more severe. The phase I detoxification enzyme cytochrome P450, phase II detoxification enzyme UDP-glucuronosyltransferase and ATP-binding cassette transporter *P-*glycoproteins could be contributing to the detoxification process. More studies of the long exposure to imidazolium-based ILs on C. elegans are needed to completely characterize the toxic effect of these ILs.

*Galleria mellonella* (greater wax moth) was studied [151] as a novel in vivo model to determine the toxicity of several 1-alkyl-3-methylimidazolium chlorides ([C_n_mim][Cl] with n = 2–18) ILs. The median lethal dose (LD_50_) values decreased with the increase of the cation alkyl side-chain length until a chain length of eight carbons ([C_8_mim][Cl]), where the lowest LD_50_ value (11.7 μg·g^−1^) was observed. Then the values slightly increased until [C_16_mim][Cl] and decreased again. This “cutoff” effect (also observed on A. *fischeri* studies) probably happened due to the retention of lipophilic compounds by *G. mellonella* larvae. The less toxic IL was, as expected, the [C_2_mim][Cl] with LD_50_ = 7538.5 μg·g^−1^, suggesting negligible toxicity for this IL, while the most toxic was the [C_8_mim][Cl]. Due to *G. mellonella* sensitivity to ILs structure, this model appeared to be reliable for future evaluation of ILs toxicity.

Cancer cells have also been used for in vitro toxicity studies. The viability [152] of human cervical carcinoma (HeLa) cells, when exposed to different doses of 1-hexadecyl-3-methylimidazolium chloride ([C_16_mim][Cl]), decreased with the increase of IL concentration and exposure time. The [C_16_mim][Cl] induced oxidative stress in HeLa cells, causing a significant decrease in the SOD and glutathione (GSH) activities at higher IL concentrations. DNA damage frequency presented a steep increase after 12 h of exposure leading to an increased number of cell deaths through necrosis or apoptosis when treated with ([C_16_mim][Cl]. Hence, ([C_16_mim][Cl] can provoke genotoxicity, physiological toxicity and induce apoptosis in HeLa cells. The cytotoxicity of this IL was also examined on human hepatocellular carcinoma (HepG2) cells [153]. HepG2 cells are a typical cell model derived from hepatocyte carcinoma, presenting vital functions in genotoxic carcinogen activation/detoxification, are commonly used to study toxin-induced hepatotoxicity. In this study, [C_16_mim][Cl] also significantly inhibited cell viability in a concentration- and time-dependent way. DNA damage increased significantly with concentration increase and longer exposure times. SOD and GSH activities were significantly repressed, especially at higher concentrations, although the malondialdehyde (MDA) concentrations were significantly enhanced, confirming that this IL can provoke oxidative stress in HepG2 cells. Like the previous study, [C_16_mim][Cl] can also provoke cytotoxicity, genotoxicity, physiological toxicity and induce apoptosis in HepG2 cells.

The channel catfish ovary (CCO) cell line has also been used to evaluate ILs toxicity as reported by Radosevic et al. [154], where the cytotoxicity of fifteen choline-based ILs and deep eutectic solvents (DESs) was analyzed toward CCO cells. The most detrimental effect on cell viability was detected with choline oxalate ([Cho][O_2_C_2_O_2_]) after 72 h of exposure (EC_50_ = 1737.85 mg·L^−1^). Apart from [Cho][O_2_C_2_O_2_], at the highest concentration (2 g·L^−1^), choline glycinate ([Cho][Gly]) was the most toxic and, while choline citrate [Cho][Cit] and choline alaninate [Cho][Ala] also showed negative effects towards CCO cells, the rest of the ILs (choline malate [Cho][Mal], choline argininate [Cho][Arg], choline asparaginate [Cho][Asp], choline lactate [Cho][Lac]) barely showed any. Regarding DESs, the highest inhibition in cell growth, after being treated at the highest concentration (2000 mg·L^−1^), was about 27% with the choline chloride:malic acid (ChoCl:MA 1:1) with an EC_50_ = 1428.093 mg·L^−1^). DESs presented higher cytotoxicity than the analogs cholinium-based ILs. CCO cells were also used to determine the cytotoxicity of fourteen imidazolium-based ILs [155]. When exposed to imidazolium-based ILs, the cells decrease their viability with the increase of the ILs concentration. Incorporating an oxygenated functionalized group on the imidazolium-based ILs will theoretically decrease the toxicity of these ILs, although no significant differences in toxic effects were observed. The results also showed that the substituent structure of the cation caused a significant effect on their toxicity, indicating that the cytotoxic effects are also structure-dependent. The microscopic analysis of CCO cells treated with the ILs revealed damages in the plasma membrane, proving that the membrane integrity was lost and inducing apoptotic and necrotic changes. A preliminary QSTR-based model was developed to help to predict the toxicity of ILs.

The cytotoxicity study of 1-methyl-3-octylimidazolium bromide ([C_8_mim][Br]) on the human hepatocellular carcinoma cells (HepG2) revealed that their viability decreases with the increase of [C_8_mim][Br] concentration. The cells’ exposure to these IL can cause cell apoptosis and, following the overproduction of reactive oxygen species, inhibition of superoxide dismutase and catalase, reduction of glutathione content, and increase of the cellular malondialdehyde level. [C_8_mim][Br] can even trigger the activation of caspases (cysteine proteases that have a key role in apoptosis induction and execution) more properly, caspase-3, caspase-8, and caspase-9 in HepG2 cells [156]. The evaluation of the effect of 1-alkyl-3-methylimidazolium chloride ([C_n_mim][Cl] with n = 8, 12, 16) ILs on calf thymus DNA showed an intercalative binding between [C_n_mim][Cl] and ctDNA, and this intercalation became stronger with the increase of the cation alkyl side-chain length [157]. The evaluation of the cyto-genotoxicological effect of ILs on four different cell lines was complemented with biodegradation studies. The ILs used in this work were: 1-butyl-3-methylimidazolium bromide ([C_4_mim][Br]), 1-decyl-3-methylimidazolium tetrafluoroborate ([C_10_mim][BF_4_]), trihexyl tetradecylphosphonium dicyanamide ([P_66614_][N(CN)_2_]), benzyldimethyltetradecylammonium chloride ([BTDA][Cl]) and 1-butyl-4-methylpyridinium chloride ([C_4_mpy][Cl]) and the selected cell lines were HaCaT (human immortal keratinocyte cells), PANC-1 (human pancreatic cancerous cells), BS-C-1 (African green monkey kidney cells) and MDA-MB-231 (human breast cancerous cells). Regarding the cytotoxicity study, it was observed that the ILs with short alkyl side-chain ([C_4_mim][Br] and [C_4_mpy][Cl]) had lower toxicity (IC_50_ > 300 mg·L^−1^) against all the cell lines. While the ILs with long alkyl side-chain ([C_10_mim][BF_4_], [P_66614_][N(CN)_2_] and ([BTDA][Cl]) had a significant cytotoxicity (IC_50_ < 49 mg·L^−1^) to all cell lines. [P_66614_][N(CN)_2_] (IC_50_ ≤ 6 mg·L^−1^) and ([BTDA][Cl] (IC_50_ ≤ 0.6 mg·L^−1^) were the ILs with higher toxicity among all cell lines. Based on these results, the cytotoxicity mainly depends on the alkyl side-chain and cell type. The ILs with shorter alkyl chain, [C_4_mim][Br] and [C_4_mpy][Cl], did not cause DNA damage in the cells even at higher concentrations (10,000 mg·L^−1^) though [C_10_mim][BF_4_] causes DNA damage at 500 mg·L^−1^. MDA-MB-231 cells exhibited significant DNA damage when exposed to [P_6,6,6,14_][N(CN)_2_] and [BTDA][Cl], even at lower concentrations (5 mg·L^−1^), both of them also present high toxicity to the cellular genetic material. The lipophilic nature of [P_6,6,6,14_][N(CN)_2_] and the dicyanamide anion may be the reason for the high toxicity of this IL. The biodegradation results revealed that [C_10_mim][BF_4_] showed 21% decrease in BOD_28_, 16%, 11%, 10% and 10% decrease was observed in the case of [BTDA][Cl], [P_6,6,6,14_][N(CN)_2_], [C_4_mim][Br] and [C_4_mpy][Cl]. The low biodegradability values can be caused by the long alkyl side-chain and cationic groups. [C_4_mim][Br], [P_6,6,6,14_][N(CN)_2_], [C_10_mim][BF_4_], [BTDA][Cl] and [C_4_mpy][Cl] caused 15.5, 28.4, 84.8, 10.8 and 15.5% increase in N-demethylase activity [158].

The dermal toxicity of seven ILs was studied [159] using human keratinocyte and fibroblast cell line, 3D reconstructed human epidermis, and a full-thickness model to elucidate the processes by which damage occurs. The human keratinocyte cell line (HaCaT) and human fibroblast cell line (Hs68) were selected to evaluate the dermal toxicity of ILs. It was observed significant cytotoxicity for the 1-ethyl-3-methylimidazolium bis(trifluoromethylsulfonyl)imide ([C_2_mim][(CF_3_SO_2_)_2_N]), in both cell lines, but it was more toxic to keratinocytes than to fibroblasts. The ILs contain the [(CF_3_SO_2_)_2_N] anion, but different cations did not significantly decrease the toxicity. Higher concentrations of 1-butyl-1-methylpyrrolidinium bis(trifluoromethylsulfonyl)imide ([C_4_mpyr][(CF_3_SO_2_)_2_N]) led to a reduction in cell numbers and rounding up of cell border in the keratinocyte cell line. The increase of [C_4_mpyr][(CF_3_SO_2_)_2_N] concentration led to an increase of the reactive oxygen species (ROS) and decreased the amount of glutathione being reversed by N-acetylcysteine (antioxidant). The results involving the 3D reconstructed human epidermis revealed that [C_2_mim][(CF_3_SO_2_)_2_N] and [C_4_mpyr][(CF_3_SO_2_)_2_N] damaged the epidermis. When [C_4_mpyr][(CF_3_SO_2_)_2_N] was treated to KeraSkin-FT, its loss viability was noticeable but not excessive. The histological examination shows that [C_4_mpyr][(CF_3_SO_2_)_2_N] dilapidated the stratum corneum but was less aggressive than the positive control (sodium dodecyl sulfate).

Vraneš et al. [160] presented three novel vitamin-based ILs, choline biotinate ([Cho][Biot]), choline nicotinate ([Cho][Nicot]) and choline ascorbate ([Cho][Asc]). The toxicity of these ILs was determined on a human non-tumor cell line (normal fetal lung fibroblasts MRC-5) and H-4-II-E (rat liver hepatoma). The results indicated that [Cho][Biot] presented slightly higher cytotoxicity than its precursor biotin, while [Cho][Asc] was significantly less toxic than ascorbic acid. All the ILs were classified as nontoxic. The antibacterial activity of these ILs was also determined against *E. coli*, *P. aeruginosa*, *L. monocytogenes* and *S. aureus*. These novel vitamin-based ILs did not have any antibacterial activity against the selected bacteria. In addition to *E. coli* and *S. aureus*, Yu et al. [44] also investigated the cytotoxicity on rat C6 glioma cells (C6) and human embryonic kidney cells (HEK-293), observing again that the ILs with the [BF_4_] anion tend to be the more toxic together with [Phpi][lac], the C6 tumor cells being slightly more resistant. Incubation time (from 24 to 48 h) showed to only slightly affect the IC_50_ values.

The recent discovery of the 1 octyl 3 methylimidazolium cation ([C_8_mim]) in soils nearby a landfill waste located in the North East of England prompted the urgent study of toxic effects of [C_8_mim]-based ILs in mammalian and environmental models and their potential to trigger primary biliary cholangitis. Leitch and coworkers [161] summed up the data available for this subject, mentioning the existence of a few studies of the toxic effect of this cation and other imidazolium-based ILs in cells, mice and in a diversity of model indicators of environmental impact. However, no data were found to assess their presence in the environment or in the trophic chain leading to risks to humans. From the available data, the authors propose that this cation, and related cations/ILs, could be converted in a carboxylic acid metabolite in the human liver, being a potential substitute of the lipoic acid in the E2 component of pyruvate dehydrogenase complex and also cause oxidative stress and other problems. The lack of studies about the impact of 1 octyl 3 methylimidazolium-based ILs on human and animal organs led the same research group to evaluate the effect of 1 octyl 3 methylimidazolium chloride in mouse organs [162]. In this study, adult male C57Bl6 mice were exposed to different concentrations (0–10 mg·kg^−1^ body weight) of [C_8_mim][Cl] via 2 intraperitoneal injections (time zero and 18 h) and the effects were observed at 24 h. No evident pathological differences were observed in the heart, pancreas and brain, but several pathological changes in the kidney and liver were observed. The levels of IL accumulation were higher in kidney tissue slices than liver tissue slices. No evident necrosis of hepatocytes was observed; however, several morphological changes were apparent. The results show that the kidney was a target organ for the toxic effects of this ionic liquid along with mild cholangiopathic modifications in the liver after intraperitoneal administration. To complete the study of the toxic effects of ILs on animals, Abdelghany and coworkers [163] studied the mammalian toxicity of 1-alkyl-3-methylimidazolium chloride ILs ([C_n_mim][Cl], n = 4, 6, 8, 10). The effect of these ILs on B-13 mitochondrial function showed a time- and dose-dependent inhibition of oxygen consumption within minutes of exposure. This is a common effect observed with these imidazolium-based ILs. The increase of the alkyl chain length also increased the inhibition of oxygen consumption, promoting caspase 3/7 induction and DNA fragmentation. None of the ILs studied directly inhibited mitochondrial complexes I-IV or complex V (F0F1-ATPase). Still, the dithionite reduction and ESR spectroscopy studies indicate a one-electron reduction of oxygen in the presence of imidazolium-based ILs, indicating the ILs function as mitochondrial electron acceptors. It was also observed that the longer alkyl chain ILs were toxic in liver progenitor cells, probably due to their higher affinity with the lipid membranes (partitioning into them) combined with their reduction capability giving rise to oxidative stress. The mitochondrial oxidative phosphorylation was inhibited by the ILs studied in this work.

Acute oral toxicity in rats is an important way to evaluate the effect of compounds on the human body. Only a few of the toxicity studies published since 2015 report some data about acute toxicity of C8mim-based ILs [161,162]. However, the data presented in these studies are found at ECHA’s inventory, where most of the information available indicates that some of them were characterized as “acutely toxic orally”, but no experimental values are available [161]. Due to the importance of the acute oral toxicity for humans and the lack of data, we would recommend an effort by the scientific community in this direction to determine the real effects of the ingestion of ILs in mammalian species.

The study of enzyme inhibition assays and cell lines provides essential data to help evaluate the impact of ionic liquids on humans and animals. The enzymes commonly used in these studies are acetylcholinesterase, lipase, carboxylesterase, catalase, cellulase and adenosine deaminase.

The exposure of *Hypophthalmichthys molitrix* (silver carp) spleen at concentrations of 1.095 and 4.380 mg·L^−1^ of 1-methyl-3-octylimidazolium bromide ([C_8_mim][Br]) showed [164] that this IL inhibited the activity of SOD, CAT, GSH and glutathione peroxidase (GPx). The MDA and protein carbonyl levels increased, revealing that the exposure to [C_8_mim][Br] induced oxidative stress. A long period of exposure to this IL could stimulate swelling in the fish spleen. The lysozyme activity and complement 3 and immunoglobulin M content are modified when exposed to [C_8_mim][Br], demonstrating that chronic exposure to this IL causes immunotoxic effects on silver carp. Moreover, exposure to this IL decreased the miR-125b levels, modified the miR-143 levels, and upregulated miR-155 and miR-21 levels, suggesting that these miRNAs may be involved in the [C_8_mim][Br]-induced inflammatory response in the fish spleen. The toxic effect of 1-methyl-3-octylimidazolium nitrate ([C_8_mim][NO_3_]) in soil microbial community and soil enzyme was presented by Zhang et al. [165]. This study revealed that the amount of IL in the soil barely changed for a 28 day period in various treatments. Significant decreases could be observed for all bacteria, fungi and actinomycetes at the highest dose of IL. [C_8_mim][NO_3_] also inhibited the diversity of the soil microbial community and the number of the genes AOA-*amoA* and AOB-*amoA*, usually at the highest dose of IL (8.0 mg·kg^−1^).

The inhibitory effect of twelve 1-alkyl-3-methylimidazolium-based ILs ([C_n_mim][X], n = 4, 6, 8 and 10 and X = [Br], [Cl], [BF_4_], [CF_3_SO_3_], [NO_3_]) was measured to investigate [166] the molecular mechanism of IL-trypsin interaction. All the ILs presented a weak suppression effect and their inhibitory power followed the order: [C_10_mim][Br] > [C_8_mim][Br] ≈ [C_6_mim][Br] > [C_4_mim][Br]; [C_10_mim][Cl] > [C_8_mim][Cl] ≈ [C_6_mim][Cl]> [C_4_mim][Cl] and [C_4_mim][Br] ≈ [C_4_mim][NO_3_] = [C_4_mim][Cl] ≈ [C_4_mim][BF_4_] ≈ [C_4_mim][CF_3_SO_3_] > [C_4_mim][CH_3_COO]. Considering only the anion effect, [C_4_mim][CH_3_COO] has the weakest inhibitory ability and presents the lowest hydrophobicity, while the ILs [C_4_mim][Br], [C_4_mim][NO_3_], [C_4_mim][Cl], [C_4_mim][BF_4_] and [C_4_mim][CF_3_SO_3_] presented similar inhibitory ability, but have greatly different hydrophobicity, suggesting that this characteristic is one variable influencing the ILs ability to inhibit the trypsin activity, but there is more to it. Thermodynamic studies point to hydrogen bonding being the main variable behind the IL-trypsin interaction. However, the hydrophobic interactions between ILs and trypsin should also be considered. The authors suggested a simple regression model where the toxicity is only related to their hydrophobicity and hydrogen bonding capability. The same laboratory studied the effects of six 1-alkyl-3-methylimidazolium-based ILs ([C_n_mim][X], n = 4, 6, 8 and X = [Br], [Cl], [BF_4_], [CF_3_SO_3_]) on the lactic dehydrogenase (LDH) activity and their molecular interaction mechanism [167]. All ILs had weak inhibition on the LDH activity following the trend: [C_4_mim][CF_3_SO_3_] > [C_4_mim][BF_4_] > [C_4_mim][Br] > [C_8_mim][Br] and [C_6_mim][Br] > [C_4_mim][Br] with the inhibitory ability increasing along their hydrophobicity. The influence of several ILs on lipase was also studied by the same group [168], where they found that the lipase activity was inhibited by the ILs and that the degree of inhibition was highly dependent on the chemical structures of the ILs. The increase of the cation alkyl chain length increased the inhibitory ability of the chloride- and bromide-based ILs. In the case of [BF_4_], [CF_3_SO_3_], perchlorate [ClO_4_] and [N(CN)_2_]-based ILs, the authors propose hydrogen bonding as the main driving force. From their results, they suggest that, again, the inhibition of ILs on lipase can be explained based on their hydrophobic character and their hydrogen bonding power. The same authors also studied the effect of five 1-alkyl-3-methylimidazolium-based ILs ([C_n_mim][X], n = 2, 4, 6 and X = [Br], [Cl], [CF_3_SO_3_]) on the hydrolysis of casein by lumbrokinase [169]. In this study, [C_2_mim][Br] had the highest catalytic reactivity: [C_2_mim][Br] > [C_4_mim][Cl] ≈ [C_4_mim][Br] > [C_6_mim][Br] ≈ [C_4_mim][CF_3_SO_3_], that is, the increase in hydrophobicity leads to decreased catalytic activity. The enzyme activity increased with increasing IL concentration. However, the enzymatic activity reaches a maximum and then the activity decreases showing a bell-shaped profile with IL concentration. At low IL concentration, a small addition of IL could significantly increase the lumbrokinase activity due to an increase of the hydrophilicity of casein by forming the IL-casein complex. At higher concentrations, the IL becomes a serious competitor for the active site of lumbrokinase through hydrogen bonding, inhibiting its activity. While the increased availability of casein is predominant at low concentrations, site sequestration is predominant at higher ones, thus obtaining a bell curve. If the initial substrate is highly hydrophilic, to begin with, we only see decreased activity with increasing concentration of ILs.

Acylase I (ACY I) has an important role in the detoxification and bioactivation of xenobiotics and other physiological functions. Therefore, an automated ACY I assay was developed to determine toxicity [170]. This assay was incorporated in a sequential injection analysis (SIA) methodology based on the deacetylation of *N*-acetyl-L-methionine, with the production of L-methionine. The toxicity values obtained showed that when comparing a non-aromatic phosphonium IL, tetrabutylphosphonium methanesulfonate ([P_4,4,4,4_][CH_3_SO_3_]), IC_50_ = 35.2 mM, with an aromatic imidazolium IL, 1-ethyl-3-methylimidazolium methanesulfonate ([C_2_mim][CH_3_SO_3_]), IC_50_ > 307 mM, a minor toxic effect was observed for the imidazolium IL. The 1-butyl-4-methylpyridinium tetrafluoroborate ([C_4_mpy][BF_4_] ]) with IC_50_ = 18.6 mM showed to be more toxic to ACY I than 1-butyl-3-methylimidazolium tetrafluoroborate ([C_4_mim][BF_4_]), with IC_50_ = 54.4 mM. It was also observed that the harmful effect of the cation side-chain length was not as prominent as observed in other studies and that the acetate anion had a negligible influence on ILs toxicity. ILs containing pyridinium and phosphonium cations, longer alkyl side-chains and tetrafluoroborate anion are more toxic on ACY I activity. An automated cytochrome c oxidase bioassay based on SIA was developed to evaluate the ILs toxicity [171]. Fifteen ILs were used to validate this bioassay with different cations, anions and alkyl side-chain lengths. Six different cations were used: imidazolium, pyridinium, pyrrolidinium, phosphonium, ammonium and choline. The results obtained showed that the ILs with non-aromatic cations phosphonium and pyrrolidinium ([P_4,4,4,4_[CH_3_SO_3_] with EC_50_ = 5.15 mmol·L^−1^ and 1-butyl-1-methylpyrrolidinium tetrafluoroborate ([C_4_mpyr][BF_4_]) with EC_50_ = 17.9 mmol·L^−1^) had lower EC_50_ than the aromatic imidazolium-based ILs ([C_2_mim][CH_3_SO_3_] with EC_50_ = 33.4 mmol·L^−1^ and 1-butyl-3-methylimidazolium chloride [C_4_mim][Cl] with EC_50_ = 20.7 mmol·L^−1^). [P_4,4,4,4_][CH_3_SO_3_] has one of the lowest EC_50_ values that can be explained by the cation head group having a quaternary “surfactant like” structure, presenting chemical and biological behaviors like the cationic surfactants. The choline cation was also less toxic than the imidazolium cation. Regarding the anion toxicity, it was observed that the BF_4_ anion has a more toxic effect.

Cunha and coworkers [172] evaluated the effect of nine ILs [C_4_mpyr][Cl], [C_10_mim][Cl], [C_4_mim][BF_4_], [C_2_mim][BF_4_], [C_2_mim][CH_3_COO], [C_4_mim][Cl], [C_2_mim][CF_3_SO_3_], [C_2_mim][CH_3_SO_3_] and [Cho][CH_3_COO] on aldolase activity, with the objective of exploring ILs as solvents for aldolase assisted organic synthesis. The implementation of aldolase catalyzed synthesis in IL media presented various advantages over the conventionally used solvents, like higher synthesis yield since their better affinity allows for improved enzyme activity compared to more toxic traditional media. Choline-based ILs and short alkyl chain imidazolium-based ILs that were associated with biocompatible anions showed to be the most promising compounds to perform aldolase-based synthesis.

In QSAR studies, Das et al. [173] used a data set of 289 ILs experimental data, a genetic function approximation algorithm and partial least-squares regression to develop a QSAR model to predict the toxic effect of ILs on rat leukemia cell line (IPC-81). The model developed had an *R*^2^= 0.869. Sosnowska et al. [174] developed six local models and one global for predicting the toxicity against the leukemia rat cell (IPC-81). It was suggested that if the developed QSAR models fulfill the OECD criteria, the global model should be applied since this model allows the prediction of the toxicity for hundreds of ILs simultaneously and is more efficient from the economic point of view. The local models allowed developing the QSAR models in a reduced number of structurally similar compounds. However, this type of model is more time-consuming. A new norm index-based QSTR was developed by Yan et al. [175] to predict the ecotoxicity of ILs on Leukemia rat cell lines (IPC-81). In this work, a new set of descriptors to characterize the interaction between anions and cations and some new atomic distribution matrices were constructed to calculate norm descriptions of ILs. The model developed had an *R*^2^ of 0.954 and an RMSE of 0.241. The obtained results suggest a good prediction accuracy and credibility of this model. Peng Zhu et al. [176] used two quantitative structure–activity relationship models to evaluate the toxicity of ILs towards the acetylcholinesterase enzyme by using multiple linear regression and an extreme learning machine (ELM). This study optimized the structure of 57 cations and 21 anions by using quantum chemistry calculations. The results show that the ELM model has a superior performance in the estimation of toxicity of ILs. In addition, acetylcholinesterase was used to study her inhibition of IL through well-defined and in silico calculated LFER descriptors [177]. This study served to understand the chemical interactions between ILs with diverse chemical structures and enzyme activity. The three different predictive LFER models were developed to explain the toxicological interactions on a molecular basis. In prediction studies, according to head groups, i.e., imidazolium and pyridinium, both had common descriptors. Still, they need additional terms, such as McGowan volume and H-bonding basicity of cation (for imidazolium-based IL) and excess molar refraction of the anion (for pyridinium-based IL) for better predictions. These QSAR models will be useful to pre-estimate enzyme inhibition of ILs for the ones who are theoretically existing, with fast, safe and cost-effective advantages.

A 3D biological functional tissue model based on a nano-hydroxyapatite, chitosan/gelatin hybrid scaffold was developed to determine the toxic effects of ILs. The cytotoxicity of 1-ethyl-3-methylimidazolium diethylphosphate ([C_2_mim][DEP]) was determined by this method and compared to a 2D model. The IC_50_ values obtained in the 3D model were 12,566 μg·mL^−1^, 9015 μg·mL^−1^ and 7896 μg·mL^−1^, for exposure times of 24 h, 48 h and 72 h, respectively. While in the 2D model, the IC_50_ values obtained for this IL were 3959 μg·mL^−1^, 2226 μg·mL^−1^ and 1884. μg·mL^−1^, for exposure times of 24 h, 48 h and 72 h, respectively. The 3D culture systems were easy to reproduce, forming an important bridge between 2D and 3D in vitro models in studying IL toxicology [178].

## 5. Conclusions

The constant need to improve and make the industrial process cleaner and more sustainable propels developing more efficient solvents and fluids. ILs have been widely studied as green alternatives to common organic solvents (due to their negligible vapor pressure) for many other applications in practice encompassing almost every modern industry. Nonetheless, over the years, it has become clear that they are not without fault even when considering only their “green” aspect since they can be very harmful to the environment. Over the last years, many advances have been made in improving our understanding of the toxicity effects of ILs on a wide variety of living organisms present in the environment. It is not feasible to assess the harm that all possible combinations of cations and anions can cause to even select model species. Still, not too broad a brush can be used to decide the damage they can inflict upon wider industrial adoption. What are then the best tools to economically and rapidly determine the influence that the presence of ILs on the environment can have? With an ever-increasing amount of experimental data available, SAR methods will better predict the toxicity of this class of fluids. Still, empirical data should ideally always accompany their introduction to the market.

It can be somewhat difficult to extract general conclusions. There are well-known effects of the length of the alkyl side-chain of the cation and its structure (pyridinium, imidazolium, piperidinium, pyrrolidinium, ammonium, cholinium, etc.), that overall point to their lipophilicity and consequent ease of penetrating the cell membrane. The stability and lipophilicity of the anion and the functional groups present on the cationic side-chains also influence the final toxicity. Regarding these, we should probably point out that there seems to be a limit, slightly dependent on species, for the increase in toxicity with the increase of the alkyl side-chain length, after which a plateau or even decreased toxicity occurs. This may be related to the bulkiness of the ions, as Weyhing-Zerrer et al. [53] suggested when they observe the results of combining imidazolium cations of varying chain length with the [(C_2_F_5_)_3_PF_3_] anion. On this subject, the effect of the anion has been comparatively less studied than that of the cation. Most amino acid anions have been tested to lower toxicity. This may be an inevitable result because so far, most variations on ionic liquids come from changing the cation, so less variety is found on the anion. Moreover, because researchers have seen how the cation is the main driver of toxicity, it makes sense to try modifying it to achieve the desired toxicity, whether higher (for antibacterial or antifungal applications) or lower depending on the application. Self-aggregation can also be the cause of decreased toxicity since it decreases the possibility of direct interaction with the protection barriers of the organisms. How dangerous an IL is to a particular species also depends on the composition and structure of these barriers that vary greatly from one organism to another. What experiments should we prioritize then? Again, as it is commonly known, when market uptake is at stake, the tests to run are those that account for where the new product is expected to be used, and thus, what organisms will contact them in regular use and in the case of spillage. International organizations and environmental agencies have standard tests to assess the danger to many model species and, of course, humans. Standard tests are not without fault, so modifications to them are well within the purview of investigators, for example, adjusting the pH of a solution to ascertain whether an observed phenomenon results from the acidic nature of the IL introduced or changing the tested species (particularly in microorganisms) to check if a known difference in the structure of the cell membrane affects the toxicity. Proposing new tests to address an identified gap in the ones currently in use also helps move this research area forward. We would like to encourage the usage of these tests together with the tools that we have seen deployed by several laboratories along this review. This will help to better understand the processes taking place at the molecular level and, consequently, better predict how new ionic liquids will affect the environment.

## Figures and Tables

**Table 1:** EC_50_ values (30 min of exposure) for the ILs towards *A. fischeri* studied by Sintra et al. [38].

Name	Acronym	EC_50_ (mg L^−1^)	Classification
(2-Hydroxyethyl) trimethyl ammonium cobalt tetrachloride	[N_1,1,1,2(OH)_]_2_[CoCl_4_]	8.9	Toxic
(2-Hydroxyethyl) trimethyl ammonium iron tetrachloride	[N_1,1,1,2(OH)_][FeCl_4_]	14.17	Moderately toxic
(2-Hydroxyethyl) trimethyl ammonium manganese tetrachloride	[N_1,1,1,2(OH)_]_2_[MnCl_4_]	48.18	Moderately toxic
(2-Hydroxyethyl) trimethyl ammonium gadolinium hexachloride	[N_1,1,1,2(OH)_]_3_[GdCl_6_]	26.12	Moderately toxic
(2-Hydroxyethyl) ethyldimethyl ammonium iron tetrachloride	[N_1,1,2,2(OH)_][FeCl_4_]	15.49	Moderately toxic
Hexyl(2-hydroxyethyl)dimethylammonium iron tetrachloride	[N_1,1,6,2(OH)_][FeCl_4_]	17.19	Moderately toxic
Hexyl (2-hydroxyethyl) dimethylammonium manganese tetrachloride	[N_1,1,6,2(OH)_]_2_[MnCl_4_]	37.51	Moderately toxic
Octyl (2-hydroxyethyl) dimethylammonium cobalt tetrachloride	[N_1,1,8,2(OH)_]_2_[CoCl_4_]	10.75	Moderately toxic
Octyl (2-hydroxyethyl) dimethyl ammonium iron tetrachloride	[N_1,1,8,2(OH)_][FeCl_4_]	17.49	Moderately toxic
Octyl (2-hydroxyethyl) dimethylammonium manganese tetrachloride	[N_1,1,8,2(OH)_]_2_[MnCl_4_]	27.96	Moderately toxic
Dodecyl (2-hydroxyethyl) dimethylammonium cobalt tetrachloride	[N_1,1,12,2(OH)_]_2_[CoCl_4_]	7.84	Toxic
Dodecyl (2-hydroxyethyl) dimethylammonium iron tetrachloride	[N_1,1,12,2(OH)_][FeCl_4_]	1.04	Toxic
Dodecyl (2-hydroxyethyl) dimethylammonium manganese tetrachloride	[N_1,1,12,2(OH)_]_2_[MnCl_4_]	0.76	Toxic
Butyl di(2-hydroxyethyl) methylammonium cobalt tetrachloride	[N_1,4,2(OH),2(OH)_]_2_[CoCl_4_]	10.41	Moderately toxic
Butyl di(2-hydroxyethyl) methylammonium manganese tetrachloride	[N_1,4,2(OH),2(OH)_]_2_[MnCl_4_]	32.26	Moderately toxic
Butyl di(2-hydroxyethyl) methylammonium gadolinium hexachloride	[N_1,4,2(OH),2(OH)_]_3_[GdCl_6_]	24.1	Moderately toxic
Hexyl di(2-hydroxyethyl) methylammonium cobalt tetrachloride	[N_1,6,2(OH),2(OH)_]_2_[CoCl_4_]	18.08	Moderately toxic
Hexyl di(2-hydroxyethyl) methylammonium iron tetrachloride	[N_1,6,2(OH),2(OH)_][FeCl_4_]	16.81	Moderately toxic
Hexyl di(2-hydroxyethyl) methylammonium manganese tetrachloride	[N_1,6,2(OH),2(OH)_]_2_[MnCl_4_]	69.54	Moderately toxic
Hexyl di(2-hydroxyethyl) methylammonium gadolinium hexachloride	[N_1,6,2(OH),2(OH)_]_3_[GdCl_6_]	34.17	Moderately toxic
Hexyl tri(2-hydroxyethyl) ammonium cobalt tetrachloride	[N_6,2(OH),2(OH),2(OH)_]_2_[CoCl_4_]	13.11	Moderately toxic
Hexyl tri(2-hydroxyethyl) ammonium iron tetrachloride	[N_6,2(OH),2(OH),2(OH)_][FeCl_4_]	5.99	Toxic
Hexyl tri(2-hydroxyethyl) ammonium manganese tetrachloride	[N_6,2(OH),2(OH),2(OH)_]_2_[MnCl_4_]	10.19	Moderately toxic
Hexyl tri(2-hydroxyethyl) ammonium gadolinium hexachloride	[N_6,2(OH),2(OH),2(OH)_]_3_[GdCl_6_]	17.52	Moderately toxic

**Table 2:** MIC values (μg·cm^−3^) obtained in work developed by Kaczmarek et al. [75].

Ionic Liquid	Bacteria										Yeasts	
	*Staphylococcus aureus* ATCC 33862	*Staphylococcus epidermidis* ATCC 12228	*Bacillus subtilis* ATCC 11774	*Enterococcus faecalis* ATCC 19433	*Micrococcus luteus* ATCC 4698	*Pseudomonas aeruginosa* ATCC 9027	*Serratia marcescens* ATCC 8100	*Proteus vulgaris* ATCC 49132	*Moraxella catarrhalis* ATCC 25238	*Escherichia coli* ATCC 8739	*Rhodotorula rubra*	*Candida albicans* ATCC 10231
[N_1,1,12,2(OH)_][glycolate]	8	31	16	8	<0.5	250	62	125	125	125	125	16
[N_1,1,12,2(OH)_][D-gluconate]	16	31	16	16	1	500	125	250	250	125	125	31
[N_1,1,12,2(OH)_][α-ketoglutarate]	8	31	16	8	<0.5	250	62	125	125	125	125	16
[N_1,1,12,2(OH)_][L-pyroglutamate]	16	31	16	8	1	250	62	125	62	125	125	16
[N_1,1,12,2(OH)_][cholate]	8	31	16	16	1	250	125	250	125	250	125	31
[N_1,1,12,2(OH)_][docusate]	125	250	125	125	250	>1000	>1000	>1000	>1000	>1000	500	125
[N_1,1,12,2(OH)_][DEHP]	16	31	31	16	31	250	62	250	62	125	125	8
[N_1,1,12,2(OH)_][Cl]	8	2	125	16	31	250	500	125	8	125	32	125
[N_10,10,1,1_][Cl]	<0.5	<0.5	0.5	<0.5	<0.5	8	4	4	< 0.5	1	1	2
[BA][Cl]	<0.5	<0.5	0.5	0.5	<0.5	16	4	8	0.5	4	4	4

**Table 3:** Passino and Smith toxicologic ranking.

Relative Toxicity	EC_50_ (μg·mL^−1^)
Super toxic	<0.01
Extremely toxic	0.01–0.1
Highly toxic	0.1–1
Moderate toxic	1–10
Slightly toxic	10–100
Practically harmless	100–1000
Relatively harmless	>1000

**Table 4:** The median lethal concentration (LC_50_) values and hazard ranking for zebrafish (*Danio rerio*) at 96 h after acute exposure to imidazolium-based ILs.

Ionic Liquid		96 h LC_50_ (µg·mL^−1^)	Hazard Ranking	Reference
Cation	Anion			
[C_2_mim]	[Cl]	2620 (2360–2830)	Relatively harmless	[93,96]
	[Br]	2970 (2760–3160)		
	[BF_4_]	2170 (1690–2540)		
	[NO_3_]	3193.5 (2912.7–3472.2)		
[C_4_mim]	[Cl]	632.8 ± 67.4	Practically harmless	[93,95,96,99]
	[Br]	897 (618–2220)		
	[BF_4_]	604.6 ± 56.2		
	[NO_3_]	867.1 (805.1–928.8)		
	[PF_6_]	550 ± 6.348		
[C_6_mim]	[Cl]	128.5 (116.1–140.0)	Practically harmless	[93]
	[Br]	164.4 (154.7–174.5)		
	[BF_4_]	120.1 (109.8–129.1)		
	[NO_3_]	144.4 (113.1–188.0)		
	[PF_6_]	146.6 (130.1–162.4)		
[C_8_mim]	[Cl]	152.3 ± 12.1	Practically harmless	[92,93,96]
	[Br]	47.8 (39.5–55.8)	Slightly toxic	
	[BF_4_]	144.0 ± 11.4	Practically harmless	
	[NO_3_]	23.1 (18.5–28.7)	Slightly toxic	
[C_10_mim]	[Br]	3.80 (2.80–5.10)	Moderate toxic	[93,96]
	[NO_3_]	4.5 (3.2–6.2)		
[C_12_mim]	[Br]	3.60 (3.00–4.20)	Moderate toxic	[93,96]
	[NO_3_]	3.7 (3.0–4.6)		

## Data Availability

Not applicable.

## Abbreviations

*A. corymbifera*	*Absidia corymbifera* (fungus)
*A.*	*Alcaligenes* (Gram-negative bacteria)
*A. fischeri*	*Aliivibrio fischeri* (Gram-negative bacteria)
*A. fumigatus*	*Aspergillus fumigatus* (fungus)
*A. hydrophila*	*Aeromonas hydrophila* (Gram-negative bacteria)
*A*. *niger*	*Aspergillus niger* (fungus)
ACY I	Acylase I
AILs	Aprotic ionic liquids
*amoA*	Ammonia monooxygenase subunit A gene
AOA	Ammonia-oxidizing archaea
AOB	Ammonia-oxidizing bacteria
*B. cereus*	*Bacillus cereus* (Gram-positive bacteria)
*B. japonicum*	*Bradyrhizobium japonicum* (Gram-negative bacteria)
*B. licheniformis*	*Bacillus licheniforms* (Gram-positive bacteria)
*B. subtilis*	*Bacillus subtilis* (Gram-positive bacteria)
BOD	Biochemical oxygen demand
BS-C-1	African green monkey kidney cells
BSLT	Brine shrimp lethality test
BTFIP	3-bis(2,2,2-trifluoroethoxy)propan-2-ol
*C. albicans*	*Candida albicans* (yeast)
*C. elegans*	*Caenorhabditis elegans*
*C. freundii*	*Citrobacter freundii* (Gram-negative bacteria)
*C. glabrata*	*Candida glabrata* (yeast)
*C. krusei*	*Candida krusei* (yeast)
*C. lusitaniae*	*Candida lusitaniae* (yeast)
*C. parapsilosis*	*Candida parapsilosis* (yeast)
*C. pyrenoidosa*	*Chlorella pyrenoidosa*
*C. tropicalis*	*Candida tropicalis* (yeast)
CAT	Catalase
CCO	Channel Catfish Ovary cell line
CHO	Chinese hamster ovary cells
CMC	Critical micellar concentration
COD	Chemical oxygen demand
*D. japonica*	*Dugesia japonica*
*D. magna*	*Daphnia magna*
DESI-MSI	Desorption electrospray ionization–mass spectrometry imaging
DMSO	Dimethyl sulfoxide
*E. coli*	*Escherichia coli* (Gram-negative bacteria)
*E. faecalis*	*Enterococcus faecalis* (Gram-positive bacteria)
E. fetida	Eisenia fetida
EC_50_	Half-maximal effective concentration
ED_100_	Preservative concentrations retarding the fungal growth by 100% than the reference
ELM	Extreme learning machine
ESH	Environmental, safety and health impact
*G. mellonella*	*Galleria mellonella*
*G. stearothermophilus*	*Geobacillus stearothermophilus* (Gram-positive bacteria)
G3TFSA	Triglyme
G4TFSA	Tetraglyme
GHS	United Nations globally harmonized system
GPx	Glutathione peroxidase
GSH	Glutathione
GST	Glutathione S-transferase
H_2_O	Water
H_2_O_2_	Hydrogen peroxide
HaCaT	Human immortal keratinocyte cells
HeLa	Human cervical carcinoma cells
HepG2	Human hepatocellular carcinoma cells
Hs68	Human fibroblast cell line
IC_50_	50% inhibitory concentration
IC_80_	80% inhibitory concentration
IC_95_	95% inhibitory concentration
ILs	Ionic liquids
IPC-81	Rat leukemia cell line
*K. pneumoniae*	*Klebsiella pneumoniae* (Gram-negative bacteria)
*L. lactis*	*Lactobacillus lactis* (Gram-positive bacteria)
*L. monocytogenes*	*Listeria monocytogenes* (Gram-positive bacteria)
*L. sakei*	*Lactobacillus sakei* (Gram-positive bacteria)
LC_50_	50% lethal concentration
LD_50_	Median lethal dose
LDH	Lactic dehydrogenase
LFER	Linear free energy relationship descriptors
LPS	Lipopolysaccharide
*M. catarrahalis*	*Moraxella catarrhalis* (Gram-negative bacteria)
MBC	Minimum bactericidal concentration
MDA	Malondialdehyde
MDA-MB-231 cells	Human breast cancerous cells
MIC	Minimum inhibitory concentration
NRRT	Neutral red retention assay
O_2_•^−^	Superoxide radicals
OECD	Organization for Economic Cooperation and Development
OH•^−^	Hydroxyl radicals
*P. aeruginosa*	*Pseudomonas aeruginosa* (Gram-negative bacteria)
*P. fluorescens*	*Pseudomonas fluorescens* (Gram-negative bacteria)
*P. mirabilis*	*Proteus mirabilis* (Gram-negative bacteria)
*P. putida*	*Pseudomonas putida* (Gram-negative bacteria)
*P. vulgaris*	*Proteus vulgaris* (Gram-negative bacteria)
PANC-1 cells	Human pancreatic cancerous cells
PILs	Protic ionic liquids
POD	Peroxidase
PSI	Photosynthetic system I
PSII	Photosynthetic system II
QSAR	Quantitative structure–activity relationship model
*R. rubra*	*Rhodoturula rubra* (yeast)
RAPD	Randomly amplified polymorphic DNA assay
RCOO	Peroxide radicals
ROS	Reactive oxygen species
*S. aureus*	*Staphylococcus aureus* (Gram-positive bacteria)
*S. cerevisiae*	*Saccharomyces cerevisiae* (yeast)
*S. enterica*	*Salmonella enterica* (Gram-negative bacteria)
*S. epidermidis*	*Staphylococcus epidermidis* (Gram-positive bacteria)
*S. marcescens*	*Serrata marcescens* (Gram-negative bacteria)
*S. obliquus*	*Scenedesmus obliquus*
*S. typhimurium*	*Salmonella typhimurium* (Gram-negative bacteria)
SEM	Scanning electron microscopy
SIA	Sequential injection analysis
SOD	Superoxide dismutase
*T. asahii*	*Trichosporon asahii* (fungus)
*T. mentagrophytes*	*Trichophyton mentagrophytes* (fungus)
*T. novellus*	*Thiobacillus novellus* (Gram-negative bacteria)
ThOD	Theoretical oxygen demand
[BA]	Alkyl-benzyldimethylammonium
[BTDA]	Benzyldimethyltetradecylammonium
[C_10_-Men-Am]	[(1*R*,2*S*,5*R*)-(−)-Menthoxymethyl] dimethyldecylammonium
[C_10_mim]	1-Decyl-3-methylimidazolium
[C_11_-Men-Am]	[(1*R*,2*S*,5*R*)-(−)-Menthoxymethyl] dimethylundecylammonium
[N_1,1,12,2(OH)_]	Dodecyl(2-hydroxyethyl)dimethylammonium
[C_12_-Men-Am]	[(1*R*,2*S*,5*R*)-(−)-menthoxymethyl] dimethyldodecylammonium
[C_12_mim]	1-Dodecyl-3-methylimidazolium
[C_14_mim]	1-Tetradecyl-3-methylimidazolium
[C_16_mim]	1-Hexadecyl-3-methylimidazolium
[C_1_mim]	1,3-Dimethylimidazolium
[C_1_OC_2_mim]	1-Methoxyethyl-3-methylimidazolium
[C_1_pi]	1-Methylpiperazinium
[(C_2_)^2^(C_1_)_2_(C_1_)_2_^3^gu]	2-Ethyl-1,1,3,3-tetramethylguanidinium
[(C_2_)_2_^2^(C_1_)_2_(C_1_)_2_^3^gu]	2,2-Diethyl-1,1,3,3-tetramethylguanidinium
[C_2_mim]	1-Ethyl-3-methylimidazolium
[C_2_mpy]	1-Ethyl-3-methylpyridinium
[OHC_2_C_1_NH_2_]	*N*-Methyl-2-hydroxyethylammonium
[OHC_2_mim]	1-(2-Hydroxyethyl)-3-methylimidazolium
[C_2_pi]	1-Ethylpiperazinium
[C_3_mpyr]	1-Methyl-1-propylpyrrolidinium
[C_4_mim]	1-Butyl-3-methylimidazolium
[C_4_mPheim]	1-Butyl-3-methyl-2-phenylimidazolium
[C_4_mpy]	1-Butyl-4-methylpyridinium
[C_4_mpyr]	1-Butyl-1-methylpyrrolidinium
[C_4_py]	*N*-Butylpyridinium
[C_6_mim]	1-Hexyl-3-methylimidazolium
[C_6_mpy]	1-Hexyl-1-methylpyrrolidinium
[C_8_mim]	1-Octyl-3-methylimidazolium
[C_8_OC_1_mim]	1-Octyloximethyl-3-methylimidazolium
[Cho]	Choline
[DBNH]	1,5-Diazabicyclo(4.3.0)non-5-enium
[DMEEEAH]	*N,N*-Dimethyl-2-(2-ethoxyethoxy)ethylammonium
[DMEtAH]	*N,N*-Dimethylethanolammonium
[DMHEEAH]	*N,N*-Dimethyl-2-(2-hydroxyethoxy)ethylammonium
[HOC_2_mim]	1-Hydroxyethyl-3-methylimidazolium
[HOC_3_mim]	Hydroxypropylmethylimidazolium
[MOxa]	Methyloxazolinium
[N_1,1,1,1_]	Tetramethylammonium
[N_1,1,1,16_]	Trimethylhexadecylammonium
[N_1,1,1,2(OH)_]	(2-Hydroxyethyl)trimethylammonium
[N_1,1,1,8_]	Trimethyloctylammonium
[N_1,1,12,2(OH)_]	Dodecyl(2-hydroxyethyl)dimethylammonium
[N_1,1,18,18_]	Dimethyldioctadecylammonium
[N_1,1,2,2(OH)_]	(2-Hydroxyethyl)ethyldimethylammonium
[N_1,1,6,2(OH)_]	Hexyl(2-hydroxyethyl)dimethylammonium
[N_1,1,8,2(OH)_]	Octyl(2-hydroxyethyl)dimethylammonium
[N_1,4,2(OH),2(OH)_]	Butyldi(2-hydroxyethyl)methylammonium
[N_1,6,2(OH),2(OH)_]	Hexyldi(2-hydroxyethyl)methylammonium
[N_10,10,1,1_]	Didecyldimethylammonium
[N_4,4,4,4_]	Tetrabutylammonium
[N_6,2(OH),2(OH),2(OH)_]	Hexyltri(2-hydroxyethyl)ammonium
[N_8,8,1,1_]	Dioctyldimethylammonium
[N_8,8,8,1_]	Trioctylmethylammonium
[NH_2_C_2_mim]	1-aminoethyl-3-methylimidazolium
[OHC_3_mim]	Hydroxypropylmethylimidazolium
[P_14,4,4,4_]	Tributyl(tetradecyl)phosphonium
[P_4,4,4,1_]	Tributylmethylphosphonium
[P_4,4,4,4_]	Tetrabutylphosphonium
[P_6,6,6,14_]	Tri(hexyl)tetradecylphosphonium
[P_8,4,4,4_]	Tri(butyl)octylphosphonium
[P_8,8,8,1_]	Trioctylmethylphosphonium
[Phpi]	1-phenylpiperazinium
[P_i4,i4,i4,1_]	Triisobutyl(methyl)phosphonium
[TMEEEA]	*N,N,N*-Trimethyl-2-(2-ethoxyethoxy)ethylammonium
[TMHEEA]	*N,N,N*-Trimethyl-2-(2-hydroxyethoxy)ethylammonium
[(C_2_F_5_)_3_PF_3_]	Tris(pentafluoroethyl)trifluorophosphate
[(CF_3_SO_2_)_2_N]	Bis(trifluoromethylsulfonyl)imide
[3AT]	3-Amino-1,2,4-triazolate
[Ala]	Alaninate
[Arg]	Argininate
[Asc]	Ascorbate
[Asp]	Asparaginate
[BF_4_]	Tetrafluoroborate
[Biot]	Biotinate
[Br]	Bromide
[Bt]	Benzotrizolate
[C(CN)_3_]	Tricyanomethanide
[C_2_H_5_COO]	Propionate
[C_2_H_5_OSO_3_]	Ethylsulfate
[C_3_H_7_COO]	Butyrate
[C_4_H_9_COO]	Pentanoate
[C_5_H_11_COO]	Hexanoate
[C_7_H_5_O_2_]	Benzoate
[C_9_H_19_COO]	Decanoate
[CF_3_SO_3_]	Trifluoromethanesulfonate
[CH_3_COO]	Acetate
[CH_3_OSO_3_]	Methylsulfate
[CH_3_SO_3_]	Methanesulfonate
[CH_3_SO_4_]	Methylsulfate
[Cit]	Citrate
[Cl]	Chloride
[ClO_4_]	Perchlorate
[CoCl_4_]	Cobalt tetrachloride
[DEP]	Diethylphosphate
[DMP]	Dimethylphosphate
[DMSIP]	Dimethyl-5-sulfoisophthalate
[docusate]	Docusate
[EtOPr]	3-Ethoxypropionate
[FCH_2_COO]	Fluoroacetate
[FeCl_4_]	Iron tetrachloride
[GdCl_6_]	Gadolinium hexachloride
[Gly]	Glycinate
[glycolate]	Glycolate
[H_2_PO_4_]	Dihydrogen phosphate
[HSO_4_]	Hydrogen sulfate
[I]	Iodide
[Lac]	Lactate
[Lau]	Laurate
[Mal]	Malate
[Md]	Mandelate
[MDEGSO_4_]	2-(2-Methoxyethoxy)ethylsulfate
[MnCl_4_]	Manganese tetrachloride
[MnCl_4_]	Manganese tetrachloride
[N(CN)_2_]	Dicyanamide
[Nicot]	Nicotinate
[NO_3_]	Nitrate
[O_2_C_2_O_2_]	Oxalate
[OH]	Hydroxide
[Ol]	Oleate
[PF_6_]	Hexafluorophosphate
[Phe]	Phenylalanine
[Pro]	Prolinate
[Pyr]	Pyrithione
[SCN]	Thiocyanate
[Ser]	Serine
[Tau]	Taurine
[TMPP]	Bis(2,4,4-trimethyl)phosphate
[TOS]	Tosylate
